# Microstructural architecture of the bony scutes, spine, and rays of the bony fins in the common pleco *(Hypostomus plecostomus)*

**DOI:** 10.1080/23144599.2024.2374201

**Published:** 2024-09-04

**Authors:** Hanan H. Abd-Elhafeez, Diaa Massoud, Mohammed S. Mahmoud, Nada Abdellah, Abdallah S. Salah, Nor-Elhoda Mohamed, Mennatallah Ali Abdelrhman Sayed, Mohamed Shaalan, Catrin S. Rutland, Alaa Sayed Abu-ELhamed, Soha A. Soliman, Fatma El-Zahraa A. Mustafa

**Affiliations:** a Department of Cell and Tissues, Faculty of Veterinary Medicine, Assiut University, Assiut, Egypt; b Department of Biology, College of Science, Jouf University, Sakaka, Saudi Arabia; c Department of Zoology, Faculty of Science, Fayoum University, Fayoum, Egypt; d Department of Histology, Faculty of Veterinary Medicine, Sohag University, Sohag, Egypt; e Department of Histology and Anatomy, School of Veterinary Medicine, Badr University in Assiut, New Nasser City, Egypt; f Institute of Aquaculture, University of Stirling, Stirling, UK; g Department of Aquaculture, Faculty of Aquatic and Fisheries Sciences, Kafrelsheikh University, Kafrelsheikh, Egypt; h Faculty of Science, Biomedicine Branch, University of Science & Technology, Zewail, Egypt; i Faculty of Medicine, King Salman University, Sina, Egypt; j Department of Pathology, Faculty of Veterinary Medicine, Caio University, Giza, Egypt; k Polymer Institute, Slovak Academy of Sciences, Bratislava, Slovakia; l School of Veterinary Medicine and Science, University of Nottingham, Nottingham, UK; m Department of Respiratory Therapy, Faculty of Applied Medical Sciences, Jazan University, Jazan, Saudi Arabia; n Department of Histology, Faculty of Veterinary Medicine, South Valley University, Qena, Egypt

**Keywords:** *Hypostomus plecostomus*, light microscope, stereoscope, scanning electron microscope, bony scutes, bony fins

## Abstract

Studying scute and fin morphology are advantageous approaches for phylogenetic identification and provide information on biological linkages and evolutionary history that are essential for deciphering the fossil record. Despite this, no prior research has precisely characterized the histological structures of scutes in the common pleco. Therefore, this research investigated the microstructure and organization of bone tissue within the dermal skeleton, including the scutes and fins, in the common pleco, using light microscopy, stereomicroscopy, and scanning electron microscopy. The dermal scutes were organized in a pentagonal shape with denticular coverage and were obliquely aligned with the caudal portion pointing dorsally. The dermal scutes consisted of three distinct portions: the central, preterminal, and terminal portions. Each portion comprised three layers: a superficial bony plate, a basal bony plate, and a mid-plate. Both the superficial and basal bony plates were composed of lamellar bone and lamellar zonal bone, whilst the mid-plate consisted of secondary osteons and woven bone. In the terminal portion, the superficial and basal bony plates became thinner. The pectoral fin consists of spines and rays composed of lepidotrichium (two symmetrical hemi-rays). The spine contained centrifugal and centripetal lamellar and trabecular bones. A centripetal fibrous bone was implanted between the lamellar bones. Besides being oriented in a V shape, the hemi-rays were also composed of thin centrifugal and centripetal lamellar bones and trabecular bones. A fibrous bone was identified between the centrifugal and centripetal bones. The trabecular bone and lamellar bone were made up of bone spicules.

## 1. Introduction

The skeleton provides a variety of functions, including protection, flexibility, structural reinforcement and support, and the synthesis of haemopoietic tissue. The structural and biological organization of the skeletal tissue is similar in mammals and birds, the endoskeleton is composed of bone and cartilage [1–3]. Reptiles and fish, have distinct endo- and exoskeletons [4,5], their endoskletons are usually bone and cartilage, and fish have a specialized dermal skeleton consisting of scales, denticles, and scutes [4]. Investigating the characteristics of various species’ dermal skeletons is essential for phylogenetic identification, understanding the relationships between species, and piecing together evolutionary biology [5].

*Hypostomus plecostomus* is a tropical armoured catfish (*Siluriformes: Loricariidae*) whose native habitat is South America [6]. *Loricariidae* ranks fifth based on the number of species among vertebrate families, as it includes a high diversity of neotropical fish species [7,8]. Due to the scarcity of fossils from this family, it is challenging to track its evolutionary history. For instance, both closely related and unrelated armoured catfishes have tooth-like structures in their integumentary systems that resemble those of early vertebrates [9]. The presence of two longitudinal series of dermal plates on the flanks distinguishes the *Callichthyidae* armoured catfishes from all other *Siluriformes* [10]. Importantly, the family now has roughly 200 recognized species spread across eight genera [10,11]. *Loricariidae* includes benthic fishes, which have many rows of scutes and copious denticles (armoured). This benthic habitat of armoured *Loricariidae* is shared with scaleless fishes, supporting the hypothesis that lineages of fish with scutes have emerged from scaleless taxa [12].

The osteoderms or dermal scutes are a special type of dermal skeleton composed of bony tissue which develop in the dermis to form scales or plates. These scutes are found in various species, including reptiles, amphibians, dinosaurs, placodonts, aetosaurs, phytosaurs, and hupehsuchians, but osteoderms are only present in few mammalian species [13,14]. Scutes likely emerged independently in different lineages of unrelated fish taxa, however, once they had been established, they did not manifest secondary losses in most cases, thus indicating their evolutionary advantage [12,15]. Several different types of dermal skeleton are present in aquatic species. Fish scales are high density, tough yet flexible mineralized structures located in the inner dermal layer of the skin. Scutes may be considered as a particular type of fish scale arising from an ossification process that leads to the formation of bone-like structures in the skin dermis [16]. The evolution of most fishes’ scales has shifted away from thick armour plates towards smaller, more flexible scale assemblies, and several fish lineages have re-evolved armour in the form of scutes [16–18].

Osteoblasts are specialized cell type that originated from the neural crest and are responsible for the formation of osteoderms [5]. It has been hypothesized [19] that the two primordial osteogenic and odontogenic components of the ancient rhombic scales were the basis for all subsequent forms of the dermal fish skeleton. Different forms eventually emerged as a result of the interaction between these two primitive stages. Primary actinopterygian ganoid scales developed from the elimination of the odontogenic component, resulting in the formation of scutes in some lineages [19]. During the 500 million years of vertebrate evolution, the initial components of the dermal skeleton were lost, reduced, or profoundly altered thereby the comparative studies based purely on external morphology have not always allowed us to reveal homologies between the currently existing components and to distinguish true homology from homoplasy (defined as similarity not resulting from common ancestry) [20]. This emphasizes the need for structural knowledge, preferably at the tissue level, to identify the similarly formed dermal skeletal components and attempt to track their evolutionary history. Structural homoplasy can occur in tissues. For example, elasmodine in the elasmoid scale and lamellar bone both have a lamellar collagenous matrix, while dentine and bone both have woven-fibred collagen. In the present study, we compared cell and tissue organization in immature fish to provide evidence of a shared tissue genesis. These characteristics may enable us to ascertain the evolutionary links between the dermal skeletons of vertebrates. This publication also describes woven and lamellar bone and the bony spiracles. In brief woven bone (sometimes referred to as primary bone) has disorganized collagen fibril arrangement, is usually present in embryonic or younger animals or during wound healing and provides greater flexibility than lamellar bone. Lamellar bone, sometimes referred to as secondary bone, is mature bone remodelled from woven bone, forms osteons and has highly organized collagen orientation (into layers, or lamellae) and is therefore able to withstand greater stress than woven bone [21]. Bony spicules, sometimes called bone spurs, are generally small, sharp fragments of bone which often become trabecular bone [22].

Importantly, to our knowledge, no prior research has precisely characterized the histological structures of scutes, making this an area ripe for further investigation [23]. Previous studies have only investigated figures that displayed gross features [12,24,25] and scanning electron microscopy [26–28]. It is difficult to detail the exact structure of the bony scutes and fins using histochemically-dyed paraffin and semithin sections. Others have focused on the carapace of turtles and reptiles and mainly highlighted the general architecture of the osteodermal structures (such as bone plates) without microscopic details. Consequently, this study aimed to investigate the microstructure and organization of bone tissues of the dermal skeleton, including scutes and fins, of the common pleco (*Hypostomus plecostomus*) using a light microscope (LM), stereomicroscope, and scanning electron microscope (SEM). Moreover, the study introduces the phylogenetic homologies in other species and the significance of these structures adapting to the aquatic environment of the pleco and other species.

## 2. Materials and methods

### 2.1. Ethical approval and samples

**Figure 1.**
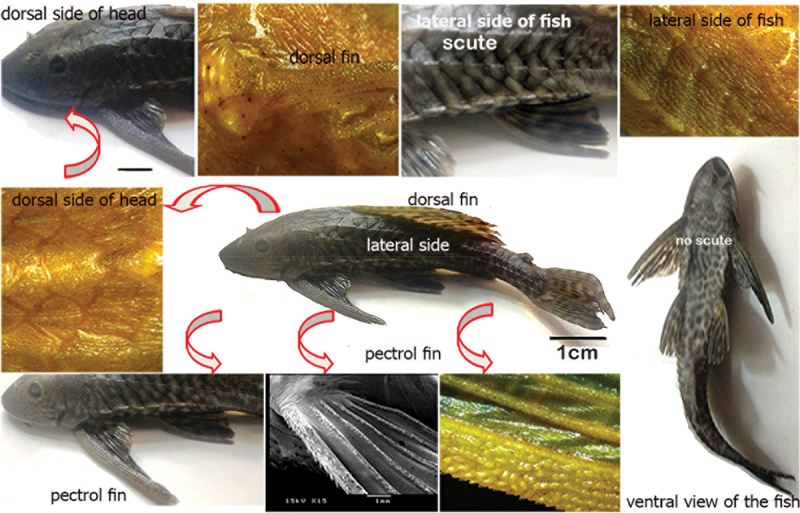
Demonstration of the structurally investigated fields of common pleco fish with general view of scutes covering the body.

The ethics committee of Kafrelsheikh University, Egypt, gave ethical approval for this study. All methods were performed in accordance with the local experimental care and used guidelines approved by the institutional animal care committee according to national guidelines and regulations (approval number: IAACUC-KSU-2022-26). Ten common pleco fish (*H. plecostomus*) were purchased from commercial providers in Assiut city, Egypt, and delivered to the wet laboratory in Assiut University. The standard body length of each fish was measured from the tip of the snout to the caudal peduncle. The immature fish, measuring 12–25 cm in length, were anaesthetized with benzocaine (4 mg/L). Half of the samples (five fish) were examined using light and stereomicroscopes, three fish were used for scanning electron microscopy, and two fish were used for semi-thin section examinations, the general anatomy of the fish is shown in Figure 1.

### 2.2. Light microscopy and staining

Three whole fish were fixed in Wrobel – Moustafa fixative for 24 h [29–31] (40 mL 25% paraformaldehyde, 125 mL of phosphate buffer (0.2 M, pH 7.4); 37.5 mL saturated picric acid; 0.5 mg of calcium chloride; and 1.25 mL of 25% glutaraldehyde; water was added to make up the total volume to 250 mL). The samples were decalcified using a mixture composed of 20 mL of 80% nitric acid, 20 mL of 10% neutral-buffered formalin, and 160 mL of distilled water for 6 weeks, with solution changes every 5 days. The samples were decalcified using a mixture composed of 20 mL of 80% nitric acid, 20 mL of 10% neutral-buffered formalin, and 160 mL of distilled water [32,33].

The decalcified and fixed specimens were dissected into three parts, then dehydrated in an ascending sequence of ethanol: three 70% ethanol baths, followed by 80%, 90% and two 100% ethanol baths. After clearing in three xylene baths, the samples were embedded in melted paraplast paraffin (Sigma-Aldrich™, USA) at 65°C in an oven (See Tables 1 and 2). The paraffin blocks were sectioned longitudinally and transversely into 4–5 µm thick sections using a Reichert® microtome (Leica RM 2155™, Germany) and placed on glass slides, which were dried overnight at 40°C in a WQ-88® incubator (Weiqian™, China).

**Table 1.** Paraffin processing and embedding technique, buffer components.

Process		Time
Procedures for dehydration		
Alcohol 70% I		24 hours
Alcohol 70% II		24 hours
Alcohol 70% III		24 hours
Alcohol 80%		1 hour
Alcohol 90%		1 hour
Alcohol 100%		15 minutes
Alcohol 100%		15 minutes
Procedures for Clearing		
Xylene I		2 hours
Xylene II		2 hours
Xylene III		2 hours
Embedding		
Praraplast I		2 hours
Paraplast II		2 hours
Paraplast III		2 hours
Na-Phosphate buffer (0.1 M, pH 7.4)	Solution A	
	Na_2_HPO_4_ 2 H_2_O	17.02 g
	Distilled water	600 ml
	Solution B	
	NaH_2_PO_4_ H_2_	6 g
	Distilled water	200 ml
	Using solution	
	Solution A	580 ml
	Solution B	219 ml
Tris-HCL buffer pH 7.4	Solution A	
	Tris	2.42 g
	Distilled water	100 ml
	Solution B	
	HCL	1.7 ml
	Distilled water	100 ml
	Using solution	
	Solution A	25 ml
	Solution B	20.7 ml
	Distilled water	Complete until 100 ml

The following conventional histological and histochemical stains were used to identify structures: Harris’ haematoxylin and eosin (H&E) stain; periodic acid – Schiff (PAS); Crossomon’s trichrome stain [34]; Mallory’s triple trichrome stain [35]; a mixture of safranin O/fast green [36]; Van Gieson stain [37] and a Grimelius silver nitrate stain [33]. All staining procedures were cited in Bancroft’s Theory and Practice of Histological Techniques [33] and components and techniques are described in the Supplemental Tables 1-4. A Leitz Dialux microscope (Leitz™, Germany) was used to examine the stained sections, and photomicrographs were captured using a Canon digital camera (Canon Powers Hot A95, Canon™, UK).

### 2.3. Stereomicroscopy investigations for identification of the surface features of the bony scutes and the surface features of rays of the fin

Two fish were used for stereo-microscopical examinations and fixed in Bouin’s fixative solution [61] (75 mL of saturated picric acid, 25 mL of formalin 40%, and 5 mL of glacial acetic acid) for 24 h. Then immersed in 70% ethanol alcohol and photographed under a binocular stereomicroscope.

### 2.4. Light microscopy investigations using toluidine blue stained semi-thin sections

For semi-thin sectioning, Karnovsky fixative (10 mL of 25% paraformaldehyde, 10 mL of 50% glutaraldehyde, 50 mL of phosphate-buffered saline, and 30 mL of distilled water) [38] was used at 4°C overnight. Small pieces of tissue (2.0–3.0 mm in length) were collected from different areas of the skin for semi-thin section preparations. Thereafter, decalcification of sections was performed at 4°C using 10% ethylene diamine tetra-acetic acid (EDTA; in 0.1 M Tris/HCl buffer, pH 7.4 [32]), the EDTA solution was changed every 5 days. After decalcification, the sections were washed four times for 15 min in 0.1 M sodium phosphate buffer saline (pH 7.2; Table 1 shows the components of the buffers). All components of the buffers were cited in Bancroft’s Theory and Practice of Histological Techniques [33].

The sections were then processed according to the methods previously described [32,39]. Samples were rinsed four times for 15 min in 0.1 M sodium phosphate buffer (pH 7.2), then post-fixed for 2 h at 4°C in 1% osmic acid in 0.1 M sodium phosphate buffer. Next, the samples were rinsed three times in 0.1 M phosphate buffer for 20 min (pH 7.2). The samples were dehydrated using a mixture of ethanol and propylene oxide. Samples were dehydrated using ascending grades of ethanol 50% for 30 min, 70% overnight, 90% for 30 min, 100% I for 30 min, and 100% II for 60 min. Resin embedding was conducted using propylene oxide (Merck, Darmstadt, Germany) for 30 min, Epon: propylene oxide (about a 1:1 ratio) for 30 min, and then Epon (5 mL of Epon 812 (Polysciences™, Eppelheim, Germany) was added to 5 mL of Araldite and 12 mL of DDSA) for 3 h and incubated at 60°C. Polymerization of the samples was conducted using the Epon mix and an accelerator (DMP-30; 1.5%). The blocks were incubated for 3 days at the following temperatures: 60°C on the first day, 70°C on the second day, and 75°C on the third day. Toluidine blue [33] [sodium tetraborate (borax) 1 g, toluidine blue 1 g, and distilled water 100 mL] was used to stain semi-thin (1 µm) sections cut with an ultra-microtome Ultra Cut E (Reichert – Leica, Germany).

### 2.5. Scanning electron microscopy investigations for identification of the surface features of the bony scutes and fins

Initially, the samples were gently washed several times using a soft toothbrush and NaCl solution to remove mucous [32] before fixation in Karnovsky fixative [38] for 24 h. Fixation was carried out as mentioned above for the semi-thin section fixation of samples using Karnovsky fixative. After fixation, the following procedures were carried out [32]: the samples were rinsed in 0.1 M sodium phosphate (the same buffer used for fixation; Table 1 shows the components of the buffers) and post-fixed for additional 2 h at room temperature in 1% osmic acid. The materials were subsequently dehydrated in acetone and isoamyl acetate; critical point drying was performed using Polaron equipment. Finally, the samples were gold-coated and examined using a JEOL SEM (JSM-5400LV, JEOL™, Welwyn Garden city, UK) at 10 kV.

The SEM pictures were coloured using Photo Filter v.6.3.2 to visualize distinct structures within the same electron micrograph. The same approaches to colour scanning electron micrographs have been used in several previous studies [32,40–44]. CMEIAS Colour Segmentation [45], an improved computer software that can analyze colour photos by segmenting the foreground objects of interest from the background, was employed to obtain negative images. Each negative image was created using the following steps: CMEIAS Colour Segmentation was used to open the image, the option “Process” was chosen from the menu, then the option “Negative image” was selected. The same approach for obtaining the negative images have been used in several previous studies [32,46–51]. The negative images for Figures 2–22 are shown in Supplementary Figures S1–21 respectively.

**Figure 2.**
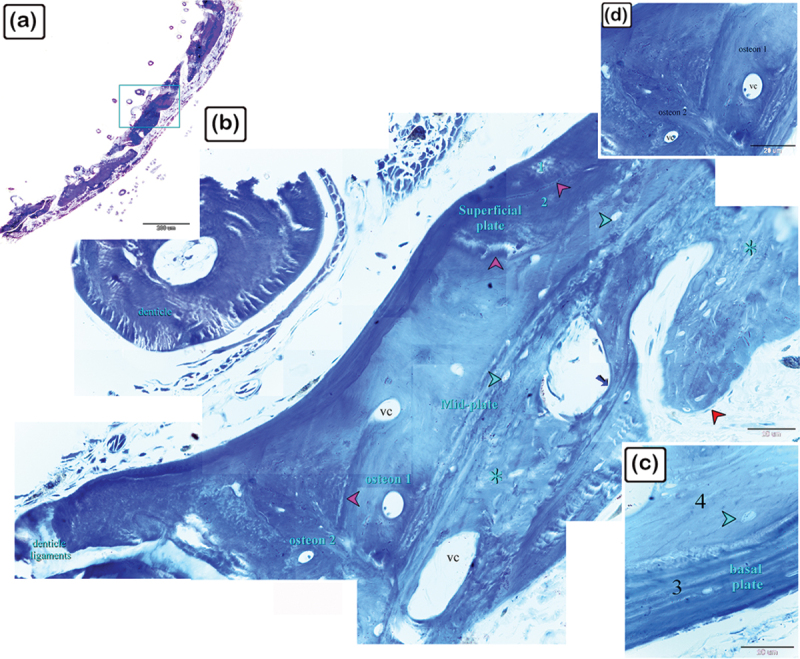
The histological structure of the central portion of the dermal scute on the dorsal side of the common pleco head. Semi-thin sections stained with toluidine blue.

(a) General view of the dermal scute, the area in the square is the central portion of the scutes. (b) The superficial bony plate consisted of two layers; the lamellar bone (1) and lamellar zonal bone (2) between which the cement line (pink arrowhead) was demarcated. The most superficial layer was lamellar bone, made up of longitudinally arranged bone lamellae. Lamellar zonal bones had concentrically organized bone lamellae around the vascular channel (vc), with multiple branches. The mid-plate was a thick bony plate consisting of secondary or mature bone osteons, composed of concentric bony lamellae surrounding the vascular cavities. Each osteon was delineated by a cement substance (pink arrowheads). The direction of the collagen fibres in the osteons (1 and 2) was perpendicularly aligned. Fibrous, fibrolamellar, or pseudo-lamellar bone filled the interstitial tissue between the osteons. This type of bone marked the mid-bony plate of the preterminal portion. Coarse collagen bundles and fibres were organized erratically in fibrous bone (*) (c) The basal plates were composed of lamellar bone and lamellar zonal bone with osteocytes (turquoise arrowheads) and osteoblasts (red arrowheads) observed. The lamellar bone (3), which consisted of longitudinally orientated bone lamellae, was the most basal layer. The bone lamellae in the lamellar zonal bone (4) were arranged concentrically around the vascular channels and the lamellar zonal bone is covered by osteoblasts. (d) The directions of the collagen fibres in the osteons (1 and 2) were perpendicularly aligned.

**Figure 3.**
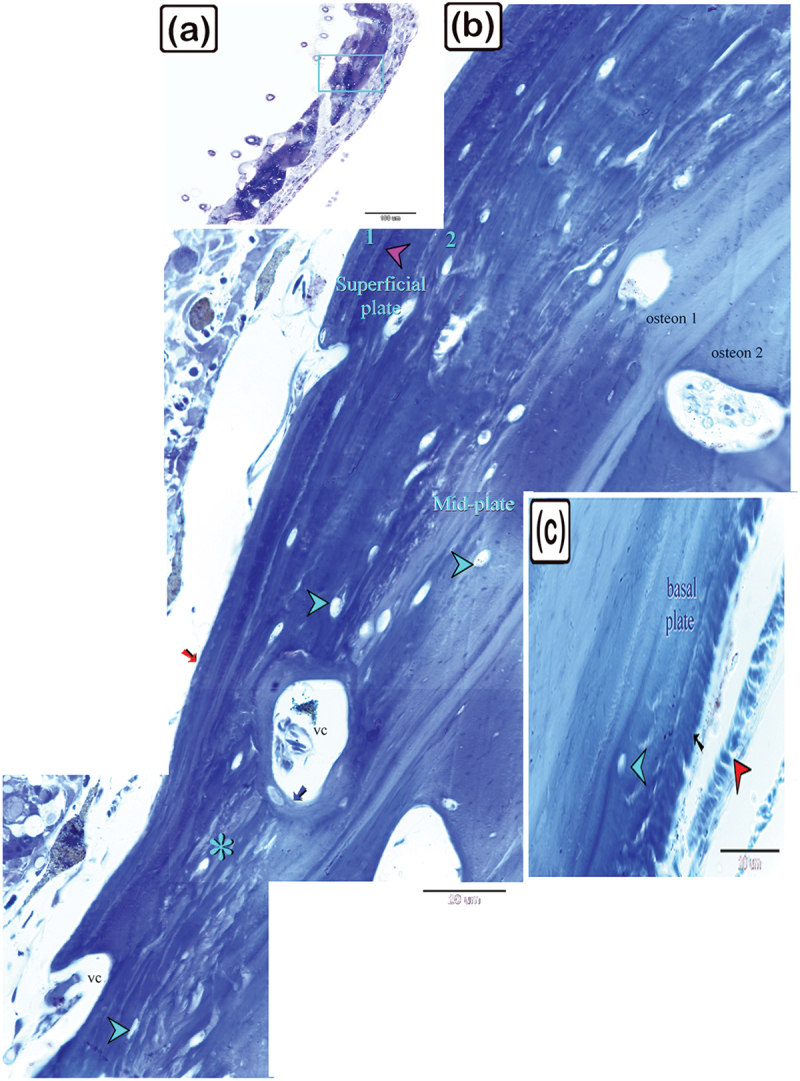
The histological structure of the preterminal portion of the dermal scute on the dorsal side of common pleco head stained with toluidine blue.

(a) General view of the dermal scute, the area within the square is the preterminal portion of the scutes. (b) The superficial bony plate consisted of two layers; the lamellar bone (1, red arrow) and the lamellar zonal bone (2) with a demarcating cement line in between (pink arrowhead). The lamellar bone was the most superficial layer, and consisted of longitudinally arranged bone lamellae. The lamellar zonal bone had concentrically arranged bone lamellae around the vascular channel (vc) with multiple branches. The mid-plate was a thick bony plate consisting of secondary or mature bone (osteon, blue arrows). The osteon was composed of concentric bony lamellae surrounding the vascular cavities (VC). Interstitial tissue between the osteons was occupied by the fibrous (*) or fibrolamellar or pseudo−lamellar bone. This type of bone marks the mid-bony plate of the preterminal portion. The fibrous bone had coarse collagen bundles and irregularly arranged fibres. Notice the osteocytes (turquoise arrowheads), osteoblasts (red arrowheads), melano-macrophage cells (red arrowheads). (c): The basal plate was composed of lamellar bone, superficial layer appeared striated (osteoblast canaliculi, black arrow). The red arrowheads refer to osteoblast and osteocytes are shown with turquoise arrowheads. Importantly.

**Figure 4.**
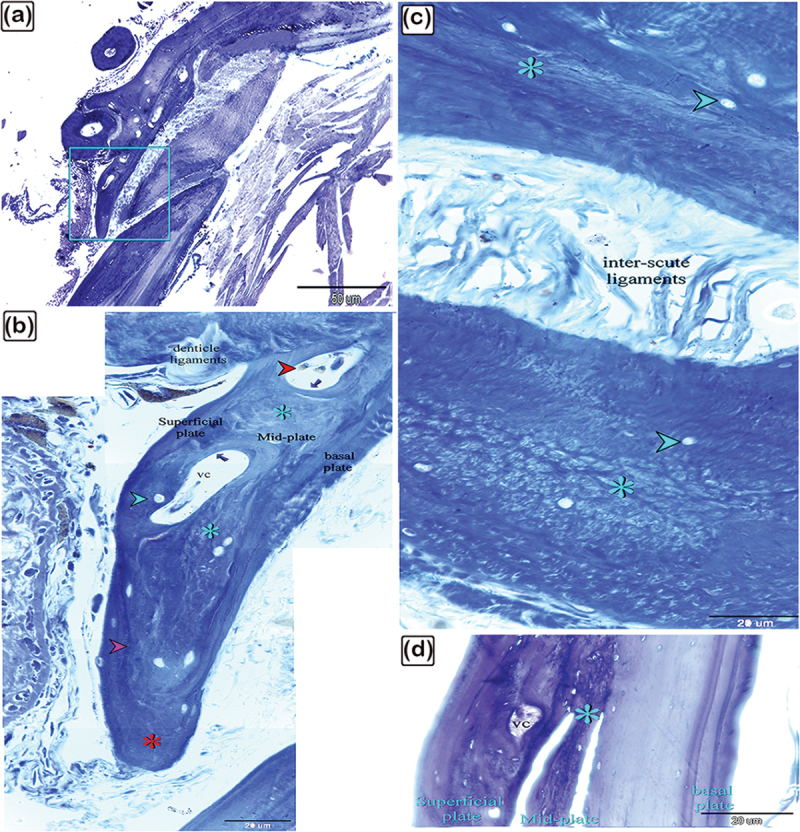
The histological structure of the terminal portion of the dermal scute on the dorsal side of the common pleco head.

Semi-thin sections stained with toluidine blue. (a): General view of the dermal scute, the square area is the terminal portion of the scutes. (b): The superficial and the basal bony plates met (red asterisk) and became thinner in the terminal portion compared to the other portions and the mid-plate also became thinner. Between the secondary osteons (blue arrows), primary or immature cellular bone containing osteocytes (turquoise arrowhead) were sometimes present. The collagen orientation in primary or immature cellular bones was irregular and woven (turquoise asterisk). The red arrowhead indicates melano-macrophage, while the pink arrowhead indicates the cement line. (c): The fibrous components of the inter-scute ligaments connected the terminal portions of two adjacent dermal scutes. *refers to fibrous bone and the turquoise arrowhead refers to osteocyte. (d): The superficial, basal, and mid-bony plates became thinner in the terminal portion compared to the other portions. The fibrous bone is marked with turquoise asterisk, while the vascular channel is marked “vc”.

**Figure 5.**
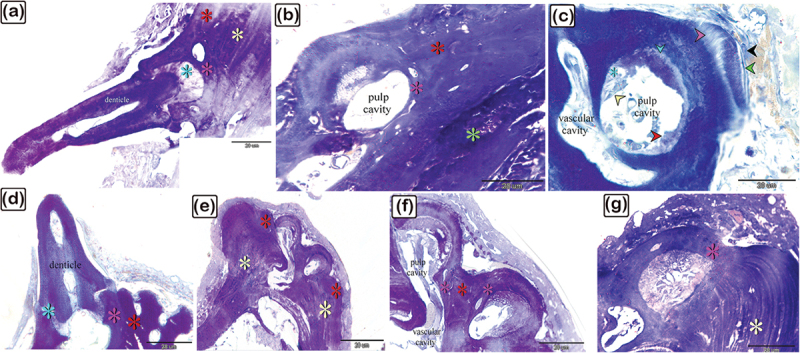
Connections between the dermal denticles and the bony scutes on the lateral side of common pleco head.

Toluidine blue-stained semi-thin sections of the dermal scute (a-c) and fin. The denticles were formed from dentin (turquoise arrowhead) and enamel (green arrowhead). The dentine is secreted by odontoblasts (red arrowhead) while the enamel was formed by ameloblast (black arrowhead). The pulp cavity of the denticle was connected to the vascular channels of bony scutes or the spines. The superficial lamellar bone (red asterisk) of the scute formed the pedicle (pink asterisk) in which the denticle ligament (blue asterisk) was attached to. Inside the pedicle, there were fibroblasts (yellow arrowhead) that formed the denticle ligament (blue asterisk). Note the osteonal lamellae and woven or fibrous bone (white asterisk).

**Figure 6.**
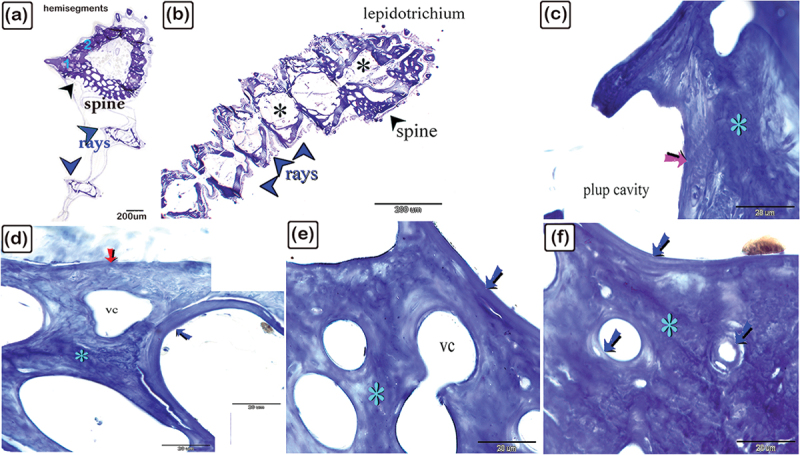
The histological structure of the spine in the dorsal fin of common pleco, semithin sections stained with toluidine blue.

(a): Cross-section of the dorsal fin showing the hemisegments (hemi-rays) of the spine (black arrowheads) and rays (blue arrowheads). The hemisegments of the spine contained centrifugal (1) and centripetal (2) trabecular bone. (b): Dorsal fin longitudinal section with lepidotrichium of the spine (black arrowhead) and rays (blue arrowheads). The pulp cavity is marked with an asterisk. (c): Thin centripetal lamellar bone (pink arrow), woven and fibrous centripetal bone (turquoise asterisk). (d-f): Thin centrifugal lamellar bone (red arrow), with the centrifugal woven or fibrous bone (turquoise asterisk), and lamellar bone (blue arrows), and vascular channels (vc).

**Figure 7.**
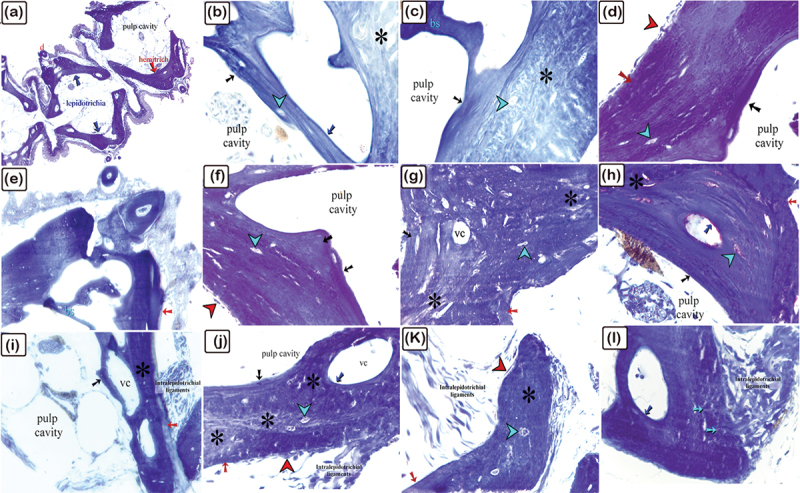
The histological structure of the rays of the dorsal fin of common pleco – semithin sections stained with toluidine blue.

(a): Longitudinal section showing the lepidotrichium (blue arrows) and rays (red arrows). The V-shaped hemisegment is identified and the letter “d” marks the denticles. (b and c): The V-shaped hemisegments have thin centrifugal lamellar bone (red arrow), centrifugal woven or fibrous bone (black asterisk), lamellar bone (blue arrows), and a pulp cavity containing nerve fibres. (d and f): Dorsal fin rays are formed in some areas by centrifugal pseudo- (red arrow) and centripetal (black arrow) lamellar bones. Inactive osteoblasts (red arrowhead), and osteocytes (blue arrowhead) were present. (g and h): Thin centrifugal lamellar bone (red arrows) and centrifugal (black arrows), woven or fibrous bone (black asterisk) in V-shaped hemisegments. The rays were made up of thin centrifugal (red arrow) and centripetal (black arrow) lamellar bones with a woven bone (turquoise asterisk) in between, with intralepidotrichial ligaments linking adjacent lepidotrichia. The bone spicules or trabeculae are marked with “bs”. (j and k): Thin centrifugal (red arrow) and centripetal (black arrow) lamellar bone. In figure (j), the woven or fibrous bone (black asterisk) was oriented in three layers and the intralepidotrichial ligaments connect adjacent lepidotrichia. (l): Notably, the intralepidotrichial ligaments continued in the centrifugal bone with the Sharpy’s fibres (double turquoise arrows). Importantly, in all figures, the osteoblasts (red arrowheads) covered the surface of the centrifugal bone, osteocytes (turquoise arrowheads), lamellar bone (blue arrows), vascular channel (vc), and woven or fibrous bone (black asterisks) were identified.

**Figure 8.**
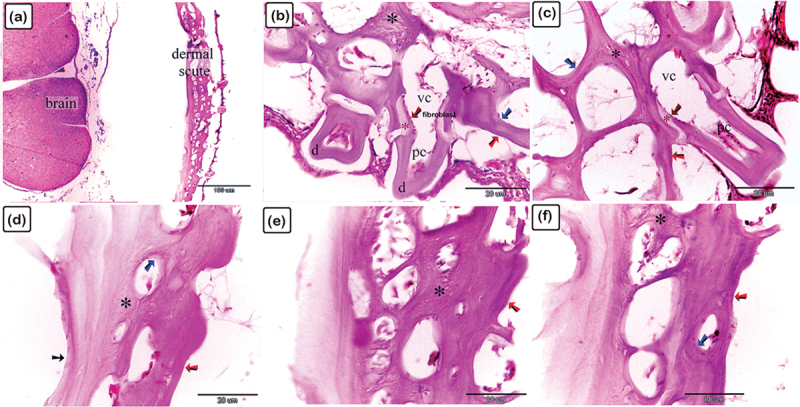
The histological structure of the dermal scute on the dorsal side of the common pleco head.

H&E-stained paraffin sections. (a): The dermal scute protected the brain. (b and c): The superficial bony plate (red arrow) was connected to the denticles (d) via the denticle ligament (brown asterisks), which is built up by the secretion of fibroblasts (brown arrow). The denticle pulp cavity (pc) continues into the dermal scutes’ vascular channels (vc). The mid-plate was formed of trabecular bone that contained fibrous bone (asterisk) and secondary osteons (blue arrow). (d-f): The dermal scute consisted of the superficial (red arrow), basal (black arrow) bony plate and secondary osteons in the mid-plate (blue arrow). The fibrous bone has been identified with an asterisk.

**Figure 9.**
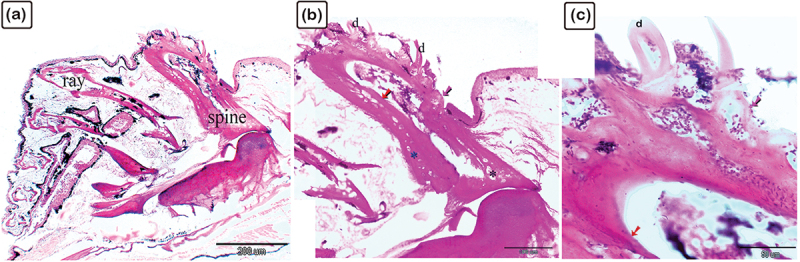
The histological structure of the spine of the common pleco dorsal fin.

H&E-stained paraffin sections. (a): The dorsal fin consisted of a spine and rays. (b and c): The spine contained centrifugal lamellar bone (pink arrow) and trabecular bone (blue asterisk), and centripetal lamellar (red arrow) and trabecular (black asterisk) bone.

**Figure 10.**
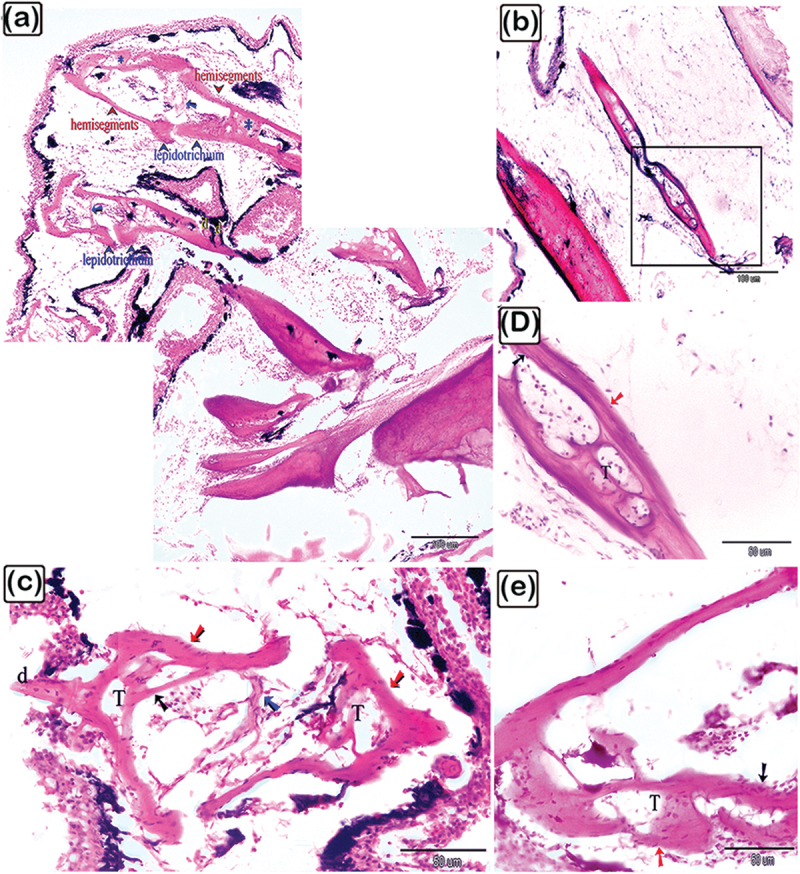
The histological structure of the rays of the dorsal fin in common pleco.

H&E-stained paraffin sections. (a-c, and e): The dorsal fin rays were made of lepidotrichium, which consisted of two hemisegments connected by intralepidotrichial ligaments (blue arrows). The denticles (d) were attached to the bony tissue of the fin ray. The hemisegments were composed of thin centrifugal (red arrow) and centripetal (black arrow) lamellar bones and trabecular bones (T). (d): The V-shaped pattern of the two hemisegments were connected by the intralepidotrichial ligaments (blue arrows) and are made up of thin centrifugal (red arrow) and centripetal (black arrow) lamellar bone and trabecular bone (T). Denticles (d) were attached to the bony tissue of the hemisegment.

**Figure 11.**
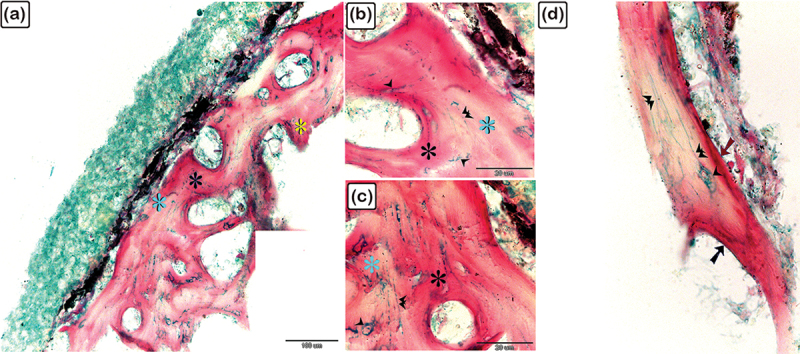
The histological structure of the common pleco scute using safranin O-fast green on the lateral side of the fish dorsal fin.

(a): A longitudinal section of the scute, with safranin-positive bone matrix present in the superficial (turquoise asterisk), basal (yellow asterisk), bony plate, and secondary osteons of the mid-plate (black asterisk). (b and c): A safranin-positive bone matrix was observed in the osteons (black asterisk) compared with the primary osteons (turquoise asterisk). Importantly, osteocytes (black arrowhead) had cytoplasmic processes (double black arrowhead) that formed a network within the bone matrix. (d): A safranin positive bone matrix was identified in the superficial (brown arrow) and basal (black arrow) bony plates underneath the periosteum.

**Figure 12.**
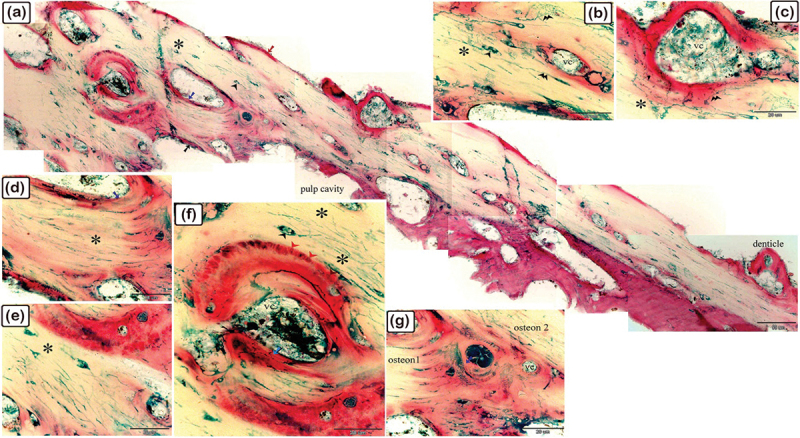
The histological structure of the spine of the common pleco dorsal fin using safranin O fast green.

(a): Longitudinal sections of the spine. Safranin positive bone matrix was observed in the centrifugal (red arrow), centripetal (black arrow), lamellar bone, and in the secondary osteons in the mid-plate (blue arrow). (b and c): With their cytoplasmic processes (double arrowhead), osteocytes (black arrowhead) built a network within the bone matrix. Lamellar bone is marked with an asterisk and the vascular channel is marked with “vc”. (d and e): Lamellar bone (asterisk) showed safranin O positive bone matrix identified in the secondary osteons of the mid-plate (blue arrow) (f): Osteocytes (black arrowhead) were observed and osteoblasts (red arrowheads) which were cuboidal in shape and aligned at the margins of the secondary osteons, secreting safranin O positive bone matrix. Note the lamellar bone (black asterisk). (g): Osteons denoted 1 and 2 were located perpendicular to each other, note the vascular channel (vc).

**Figure 13.**
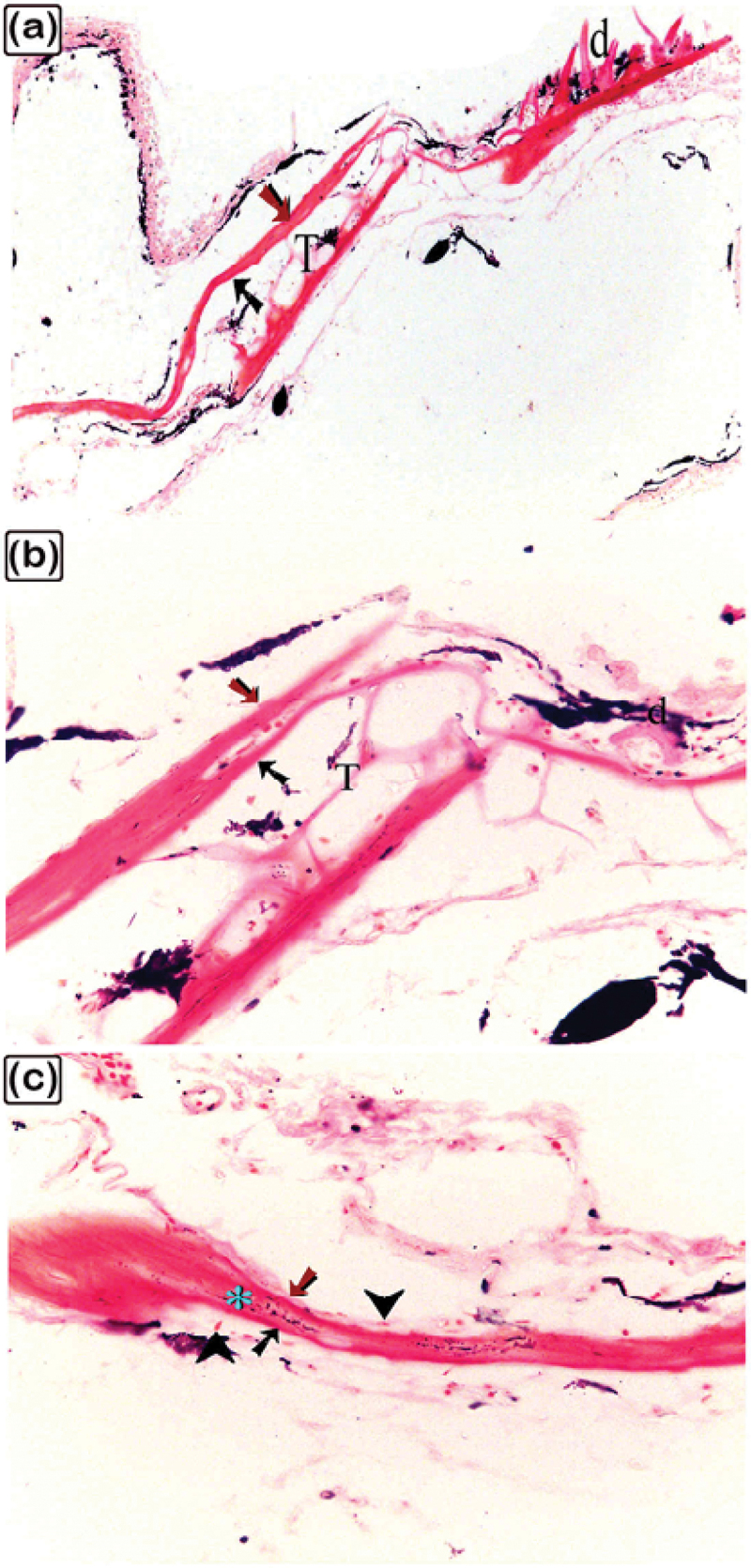
The histological structure of the longitudinal view of the hemisegment in the dorsal fin ray of common pleco using safranin O-fast green.

(a and b): Safranin O positive bone matrix hemisegments were located in the centrifugal (brown arrow) and centripetal (black arrow) lamellar bones, as well as in the trabecular bone (T). Note the denticles (d). (c): Safranin O positive bone matrix was also identified in the centrifugal (brown arrow) and centripetal (black arrow) lamellar bones, however a weaker affinity for Safranin O was detected in the deeper lamellar bones (turquoise asterisk).

**Figure 14.**
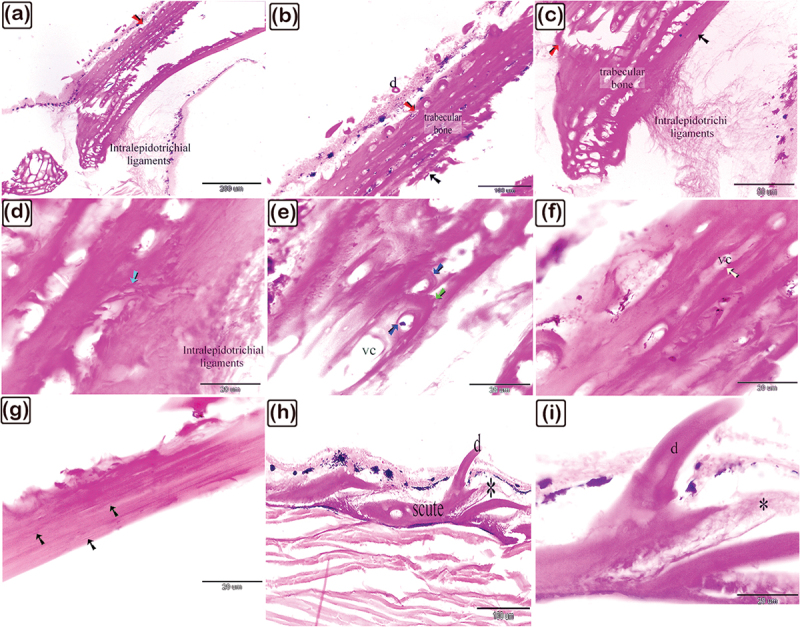
The histological structures of the fin spine and scute of common pleco fish using PAS.

Spine of the dorsal fin (a-g) and dermal scute (h, i). (a-c): PAS-positive matrix of the centrifugal (red arrow), centripetal (black arrow), and lamellar bones, note the intralepidotrichial ligaments. (d): Sharpy’s fibres (turquoise arrow) continued alongside the intralepidotrichial ligaments. (e and f): PAS-positive bone matrix (blue arrow) was observed around the vascular channels (vc, white arrow) and the secondary osteons (green arrow). (g): Osteocytes (black arrows) were located in lacunae in the lamellar bone. (h and i): All layers of the scute were PAS-positive. Note the denticles (d) and inter-scute ligaments (black asterisks).

**Figure 15.**
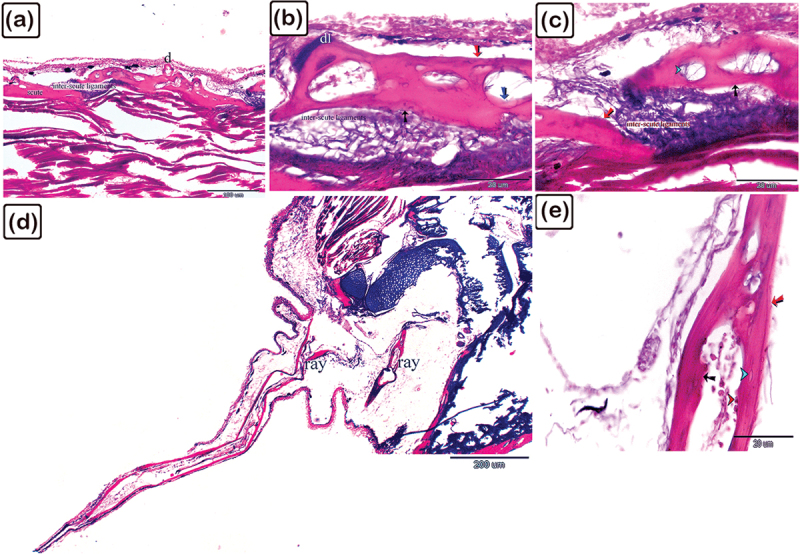
Histological structure of the scute on the lateral side of the common pleco head using the Mallory’s triple stain.

Paraffin sections of the dermal scute (a-c) and fin rays (d and e). (a-c): Longitudinal sections of the scute with inter-scute ligaments connecting the dermal scutes. The scute had a high affinity for orange G and acid fuschin. Note the superficial (red arrows) and basal (black arrows) bony plates, and the secondary osteons in the mid-plate (blue arrow). The denticles (d) were connected to the scute by the denticle ligaments (dl). Collagen fibres, which emerged from the inter-scute ligaments, penetrated the scute forming Sharpey’s fibres. (d, e) Longitudinal paraffin sections of hemisegment of the fin ray. The centrifugal (black arrow) and centripetal (red arrow) lamellar bone and trabecular bone (T) of the fin rays had high affinity for orange G and acid fuschin. Note, the red arrowhead referring to osteoblast and turquiose arrowhead referring to osteocytes.

**Figure 16.**
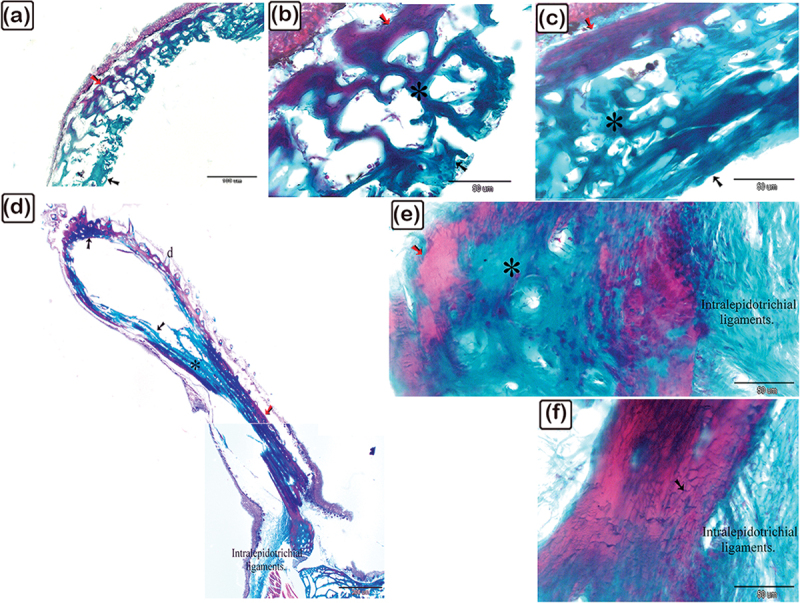
The histological structure of the spine of the pectoral fin of common pleco fish demonstrated with Crossman’s trichrome.

Paraffin sections of the scute (a-c) and pectoral fin (d-g). (a-c): Longitudinal sections of the scute. The superficial plate (red arrows) showed high affinities for orange G and acid fuschin, while the mid (black asterisk) and basal plates (black arrows) demonstrated high affinities for light green. (d): Longitudinal sections of the spine. The centrifugal (red arrow) lamellar bone revealed strong affinities for orange G and acid fuschin, while the centripetal (black arrow) lamellar bone and trabecular bone (asterisk) had high affinities for light green. (e-f): The intralepidotrichial ligaments continued as Sharpy’s fibres (black arrows). The centrifugal (red arrow) lamellar bone exhibited strong affinities for orange G and acid fuschin, while the trabecular bone (black asterisk) demonstrated a high affinity for light green.

**Figure 17.**
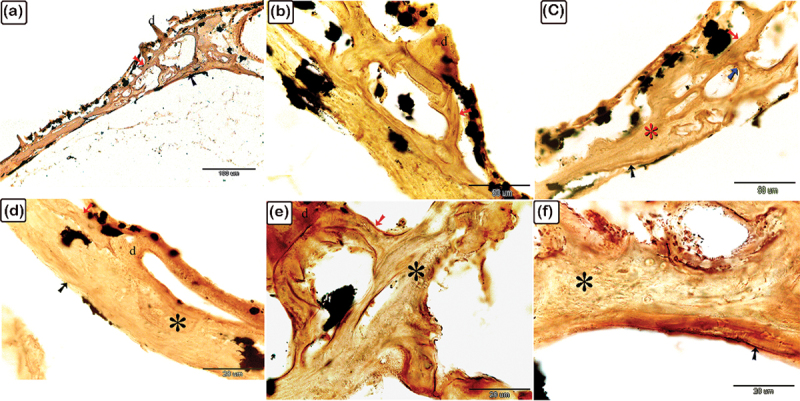
Histological structure of the spine of the dorsal fin in common pleco fish – silver stain.

(a-f): Longitudinal sections of the bony scute identifying the different layers of the bony scutes: the superficial plate (red arrows), basal plates (black arrows), and the mid-plate which consisted of lamellar bone (red asterisk), woven bone (black asterisk), and secondary osteons (blue arrow). The denticles (d) exhibited striations of the dentinal tubules.

**Figure 18.**
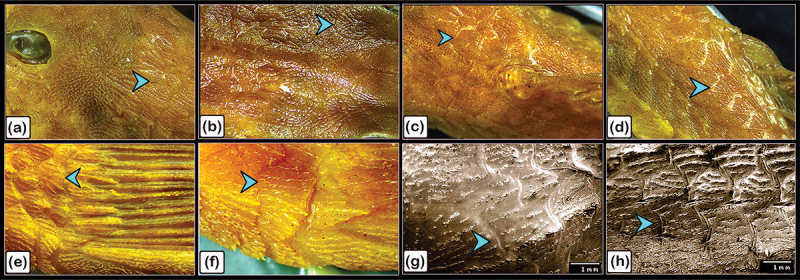
Identification of the surface features of the bony scutes in common pleco fish.

Stereoscope (a-f) and SEM (g, h) showing the dermal scutes (turquoise arrowheads) which were organized in a pentagonal shape and were covered with denticles. The scutes were obliquely aligned, with the caudal portion pointing dorsally (turquoise arrowheads). (a, b, and c) Represent the head’s dorsal side, (d) represents the fish’s lateral side, while (e and f) represent the caudal peduncle. Stereoscope image magnifications: (a-e) 6.3×; (f) 12.5 × .

**Figure 19.**
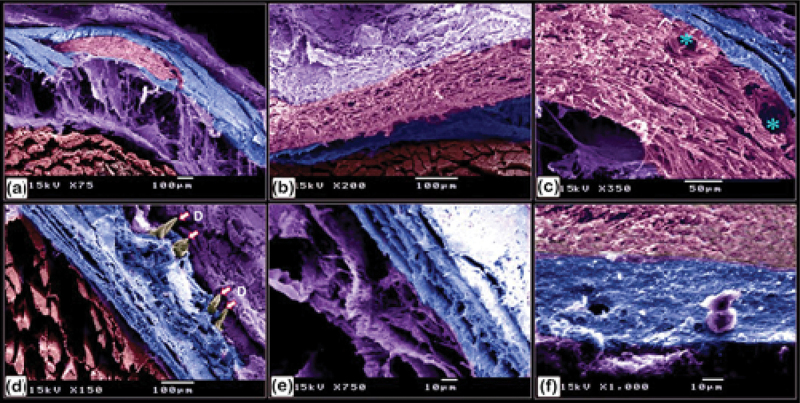
Identification of fibrillary organization of the lamellar, lamellar zonal bone, and osteonal lamellae of the scute using SEM on the lateral side of the common pleco fish.

(a-e): Lamellar (blue coloured) and lamellar zonal bone (pink coloured) comprises the superficial and the basal bony plates of the scutes. The mid-plate (pink coloured) contains secondary osteons (turquoise asterisks). The superficial and basal bony plates at the terminal portion of the scutes were made up of both lamellar and lamellar zonal bone. Notice the denticles (golden in colour marked with an arrow and D). (f): The basal bony plates of the scutes were comprised of lamellar bone (blue coloured), lamellar zonal bone (pink coloured), with plates separated by the demarcating cement line (violet coloured).

**Figure 20.**
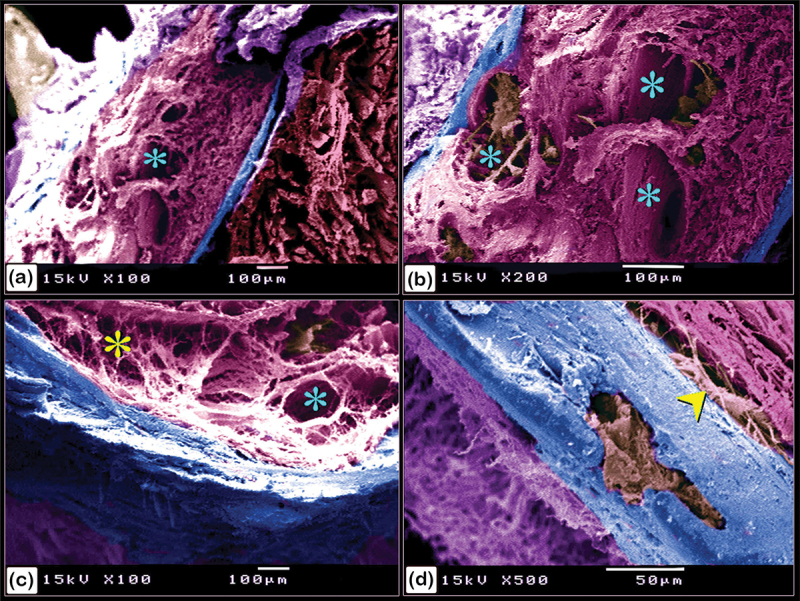
Scanning electron microscopy identification of vascular channels and fibrous bone in the common pleco scute.

(a – c): Lamellar with lamellar zonal bone (blue-coloured) comprising the superficial and the basal bony plates of the scutes on the lateral side of the fish. The mid-plate (pink-coloured) contained secondary osteons (blue asterisks). Note the osteogenic cells (brown in colour) located in the vascular channels and the fibrous bone (yellow asterisk). (d): Osteogenic cells (brown coloured) were recognized in the vascular channel of the basal plate. Note that osteocytes (yellow arrowhead) demonstrated a flattened profile and multiple cell processes.

**Figure 21.**
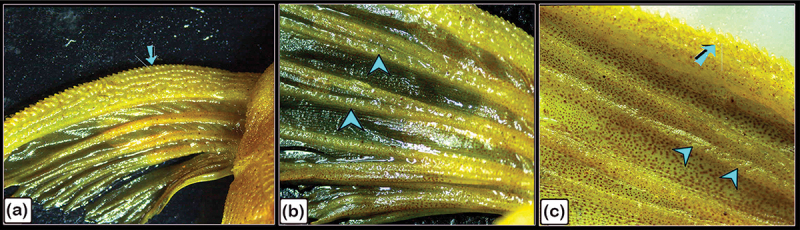
Stereoscope structure features of the pectoral fin rays in the common pleco.

The pectoral fin was composed of a single spine with numerous large declines (turquoise arrows) and fin rays covered by a web of skin and bearing small denticles (turquoise arrowheads). Magnification of stereo images: (a) 6.3×; (b) 12.5×; (c) 40 ×.

**Figure 22.**
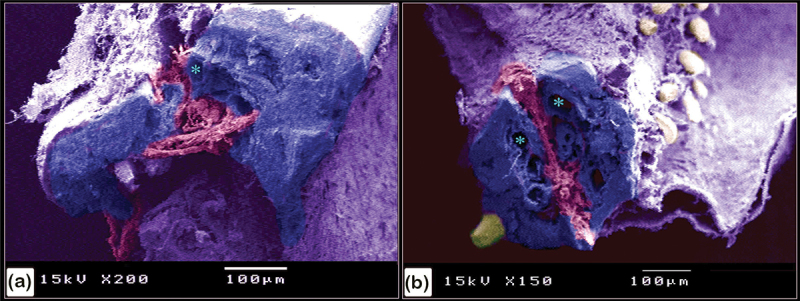
Scanning electron microscopy structure of pectoral fin rays in the common pleco fish.

(a and b):Two hemisegments (blue coloured) formed the lepidotrichium, which attached to the intralepidotrichial ligaments (pink coloured). Notice the trabecular bone (turquoise asterisks) and denticles (golden colour). Negative images of all figures are included in the supplementary material (Supplementary Figures S1-21).

## 3. Results

### 3.1. Histological structure of bony scutes (using toluidine blue on semi-thin sections)

The dermal scutes consisted of three portions: the central, preterminal, and terminal portions.

#### 3.1.1. Central portion

As demonstrated in Figure 2, the central portion of the dermal scutes consisted of the superficial bony plate, the thick bony mid-plate, and the basal bony plate. The superficial bony plate was composed of two layers: lamellar bone (the most superficial layer) and lamellar zonal bone, with a demarcating cement line in between. The lamellar bone consisted of longitudinally arranged bone lamellae and the lamellar zonal bone had concentrically arranged bone lamellae surrounding the vascular channel, which gave rise to several branches. The mid-plate was a thick bony plate consisting of mature lamellar bone (and osteons). The osteons were composed of concentric bony lamellae surrounding the vascular cavities. Each osteon was delineated by a cement substance. Primary/immature cellular bone (woven bone), which was sometimes located between the osteons (within the mature lamellar bone), had irregularly oriented collagen with a woven appearance. The basal bony plate, similar to the superficial bony plate, was composed of two layers: lamellar bone and lamellar zonal bone, with a demarcating cement line in between. As observed in the superficial bony plate, the lamellar bone was made up of longitudinally arranged bone lamellae and the lamellar zonal bone had concentrically arranged bone lamellae surrounding the vascular channel. The lamellar zonal bone was covered with osteoblasts.

#### 3.1.2. Preterminal portion

As demonstrated in Figure 3, the preterminal portion of the dermal scutes consisted of the superficial bony plate, the thick bony mid-plate, and the basal bony plate. The superficial bony plate was similar to the superficial bony plate in the central portion. The mid-plate was a thick bony plate consisting of lamellar bone and osteons. The osteons were composed of concentric bony lamellae surrounding the vascular cavities. Each osteon was delineated by a cementing substance and the direction of the collagen fibres in the osteons was perpendicularly aligned. Notably, the interstitial tissue between the osteons was occupied by fibrous or fibrolamellar bone. This type of bone marks the mid-bony plate of the preterminal portion. The fibrous bone had coarse collagen bundles and other irregularly organized fibres. The basal bony plate was similar to the basal bony plate of the central portion. Importantly, in the lamellar zonal bone, the concentric collagen fibres around two adjacent vascular channels were perpendicularly aligned to each other.

#### 3.1.3. Terminal portion

As demonstrated in Figure 4, the superficial and basal bony plates met and became thinner compared to the other portions. At this point, the terminal portion was joined to fibrous inter-scute ligament components, moreover, the mid-plate was thicker. Notably, the woven bone, observed aligned between the osteons in lamellar bone, was made up of irregularly orientated collagen with a woven appearance.

Toluidine blue staining also showed the connections between the dermal denticles and the bony scutes and fin spines. The vascular channels within the superficial bony plates continued with the pulp cavity of the dermal denticles, with the lamellar bone forming the pedicle to which the denticles’ ligaments were attached. In the pedicle, along with the odontoblasts which secrete dentin, fibroblasts were observed which build up the denticle ligament. Moreover, ameloblasts were noted which build up the enamel, and the enamel formed a thin layer on the surface of the denticles (Figure 5).

### 3.2. Histological structure of the spine and rays of the dorsal fin using toluidine blue

The dorsal fin spine consisted of lepidotrichium and was composed of two hemisegments (Figure 6(a,b)). The spine consisted of centrifugal and lamellar trabecular bone tissue, and centripetal lamellar and trabecular bone. Both the centrifugal and centripetal woven or fibrous bone were identified between the lamellar bone (Figure 6(c-f)).

The rays of the dorsal fin consisted of lepidotrichium, organized in V-shaped hemisegments. The intralepidotrichial ligaments connected the adjacent lepidotrichium. In addition, the hemisegments were distinguished by thin centrifugal and centripetal lamellar bones. The woven or fibrous bone (pseudo-lamellar bone) was identified between the centrifugal and centripetal bones. Bone spicules formed the trabecular bone, and lamellar bone was distinguished between the centrifugal and centripetal bone (Figure 7(a-i)). Inactive osteoblasts were recognized at the surface of the centrifugal bone and the intralepidotrichial ligaments continued within the centrifugal bone with Sharpey’s fibres, which penetrated the centrifugal bones (Figure 7(j-l)).

### 3.3. Analysis demonstrating the histochemical properties of the dermal tissue using H&E

H&E stain was used to identify the bony plates of the scutes. The superficial bony plate was linked to the denticles by the denticle ligament, created via fibroblast secretion, these cells were also located within the region. The vascular channels of the dermal scutes continued into the pulp cavity of the denticles. The mid-plate consisted of trabecular bone, fibrous bone, and secondary osteons (Figure 8(a-f)).

The dorsal fin consisted of the spine and rays. As observed using H&E, the spine was made up of centrifugal and lamellar trabecular bones, and centripetal lamellar and trabecular bones (Figure 9(a-c)). The rays of the dorsal fin consisted of lepidotrichium, which formed two hemisegments attached by intralepidotrichial ligaments. Besides being organized in a V-shaped pattern, the hemisegments were composed of thin centrifugal and centripetal lamellar bones and trabecular bones (Figure 10(a-e)). The denticles were attached to the bony tissue of the fin ray.

### 3.4. Histological structure of the scute using safranin-O/fast green

In the scute of the dorsal fin, safranin-positive bone matrix was identified in nascent bone areas such as the superficial bony plate, the basal bony plate, and the secondary mid-plate osteons, notably safranin-O has a low affinity for mature bones (Figure 11(a-d)). The cytoplasmic processes of the osteocytes formed a network within the bone matrix (Figure 11(b-d)).

In the dorsal fin, the centrifugal, centripetal lamellar bone, and secondary osteons of the mid-plate all demonstrated a safranin-positive bone matrix (Figure 12(a-e)). Inside the lacunae, the osteocytes had cytoplasmic processes that formed a network within the bone matrix (Figure 12(b,c); osteocytes are represented by arrowheads). Osteoblasts, which were cuboidal in shape and were aligned at the secondary osteon margins, secreted the safranin-positive bone matrix (Figure 12(f)). The adjacent osteons were perpendicularly aligned to each other (Figure 12(g)).

In the ray of the dorsal fin, the top layer of centrifugal and centripetal lamellar bone and trabecular bone, had hemisegments which were made of the safranin O-positive bone matrix. In the deeper lamellar bone, there was a weak affinity for safranin O (Figure 13(a-c)).

### 3.5. Observation of histological structures using PAS, Mallory’s triple, Crossomon’s trichrome, and silver stains

The PAS-positive matrix of the centrifugal and centripetal lamellar bones was observed in the dorsal fin spine (Figure 14(a-c)). Sharpey’s fibres continued in the intralepidotrichial ligaments (Figure 14(d)) and PAS-positive bone matrix was found around vascular channels and secondary osteons (Figure 14(e,f)). Osteocytes were present in lamellar bone lacunae (Figure 14(g)) and all of the layers within the scute were PAS-positive (Figure 14(h,i)).

The histological structures of the scute and fin rays were observed using Mallory’s triple stain. The inter-scute ligaments linked the dermal scutes together. Orange G was highly affinitive in all of the bony scute layers, including the superficial, mid-, and basal plates. The denticle ligament connected the denticles to the scute. Sharpey’s fibres were formed by collagen fibres that emerged from the inter-scute ligaments and penetrated the scute (Figure 15(a-c)). The centrifugal and centripetal lamellar bones, as well as the trabecular bone within the fin ray hemisegment, all had high affinities for Orange G (Figure 15(d,e)).

The superficial plate of the dermal scute on the pectoral fin demonstrated a high affinity for orange G, using Crossomon’s trichrome stain whereas the mid- and basal plates revealed high affinities for light green (Figure 16(a-c)). Notably, the centrifugal lamellar bone of the fin spine showed a strong affinity for orange G, whereas the centripetal lamellar bone and trabecular bone revealed strong affinities for light green. Sharpey’s fibres replaced the intralepidotrichial ligaments (Figure 16(d-g)).

A silver stain was used to identify the orientation of different layers of the bony scutes in the dorsal fin, including the superficial, mid-, and basal plates. The silver stain also differentiated between different types of bone, including lamellar bone, woven bone, and secondary osteons (Figure 17).

### 3.6. Morphological analysis of the surface features of the bony scutes and fins

A stereomicroscope (Figure 18(a-f)) and a scanning electron microscope (Figure 18(g,h)) were used to identify the surface features of the bony scutes. The dermal scutes were organized in a pentagonal shape and were covered with denticles. The scutes were obliquely aligned in a pattern whereby those in the caudal portion pointed dorsally. By observing scanned features of the scutes, the lamellar and lamellar zonal bones were comprised of the superficial and basal bony plates of the scutes. The lamellar bone and lamellar zonal bone were separated by a demarcating cement line. The mid-plate contained secondary osteons, and denticles covered the surface of the scutes (Figure 19(a-f)). Osteogenic cells were identified in the vascular channels (Figure 20(a-c)), while osteocytes with flattened profiles and multiple processes were observed in lacunae (Figure 20(d)).

The pectoral fin was composed of a single spine with large denticles whilst the fin rays were covered by a skin web displaying small denticles (Figure 21). In the pectoral fin, the lepidotrichium, which was composed of two hemisegments, was attached to the intralepidotrichial ligaments. The hemisegments were characterized by trabecular bone and covered by denticles (Figure 22).

## 4. Discussion

*Hypostomus plecostomus* is one of the ray-finned catfish and its swimming behaviour suits its function as a bottom-feeder suckerfish. The soft-rayed fin described in the present research enables slow swimming, manoeuvring between obstacles, and helps with escaping from predators whilst also enabling feeding. Plectostomus do not rely solely on their fins when swimming as they can also use their suctorial apparatus to climb vertical surfaces and to attach to the bottom enabling resistance of water flow [52] as *Hypostomus plecostomus* generally inhabits fast-flowing streams and rivers. The dermal skeleton described in this research, particularly the bony scute, potentially provides a protective function and the fact that the Plectostomus scutes were obliquely aligned in the craniocaudal direction may indicate potential functions. This orientation of the bony scute may help stream potentially harmful particles such as sand and gravel slide past the fish, thus minimizing harmful effects and damage that could be caused by these particles/stones. The existence of collagenous ligaments connecting the scutes together, as well as the hemisegments of the spine and rays, provides an efficient, mouldable protective barrier because of the flexible properties of the collagen fibres. This is supported by previous research investigating the properties of collagen [53].

In our study, we noticed the layered bone lamellae and collagen bundles in different parts of the bony scute as well as the highly mineralized enameloid in the dermal denticles, which contribute towards the defence mechanisms especially in relation to the catfish being bitten by predators. Zhu *et al*. [54] concluded that the bony surface plates protect against the piercing action of predators’ teeth. Further areas of research may include functional measurements of predation, such as those exhibited by Lowe and co-authors who researched the impact of piranha predator performance, especially bite force, against the armoured threestripe cory catfish (*Corydoras trilineatus*) [55]. It was interesting to note that the catfish in that previous study were able to withstand several piranha bites in their armoured regions, and indeed the behaviour of the catfish meant these regions were usually presented to the predator when under attack. Predation was generally only successful when unarmoured regions were bitten. Another previous study into catfish protection has also highlighted the importance of the hard armour whilst also maintaining flexibility to allow mobility [27], as also observed in our study. The covering of bony scutes on the body additionally helps explain how the pleco can adapt to living in cold water by warming its body through the vascular tissue of the marrow spaces between the scutes. The blood supply is important in the thermoregulation of the integument in fish [56].

We also discussed the structural and histochemical properties of the dermal scutes, fin spine, and the rays. The dermal scute consists of three portions: the central, preterminal, and terminal portions. Both the superficial and basal bony plates of the scute were dominated by mature bone, whereas the primary bone (also called immature, fibrous, fibrolamellar, or woven) was frequently present in the mid-plate to form the lamellar bone. To understand the lamellar organization and characteristics of the bone matrix, we conducted several histochemical techniques. PAS stains polysaccharides and safranin O/fast green identified the existence of specific types of glycoproteins, the proteoglycans essential for bone growth. Importantly, all layers of the scute were PAS-positive. Safranin O selectively stains the proteoglycans and a safranin-positive bone matrix was observed in areas of nascent bone, including the superficial and basal bony plates, and the secondary osteons within the mid-plate. Proteoglycans such as decorin and biglycan play fundamental role in fibrillogenesis and assembly of type I collagen [57–59]. These results suggest that the formation of bone lamellae of the superficial and basal bony plates probably occurs through appositional growth by secreting a bone matrix located at the periosteal surface of the bone. The formation of the osteonal lamellae of the mid-plate requires the deposition of concentric collagenous lamellae around the vascular channels that primarily establish the fibrous bone, which in turn transformed into the secondary osteon. A similar observation was reported previously [60], and it was suggested that scute formation occurs in the dermis through the osteogenic pathway by concentric ossification. The fin spine and rays demonstrated a safranin O-positive bone matrix in the centrifugal and centripetal lamellar bones, and the secondary osteons within the mid-plate of the spine, indicating areas of active bony growth. Moreover, the fin rays also exhibited a strong safranin O matrix in all layers. The dendrite/canalicular system of the osteocytes inside the bone matrix were also clearly identified by safranin O/fast green. The canalicular system was stained positively with fast green, indicating the presence of fibrous collagen. This result could be explained by work presented by Creecy and co-authors in that osteocytes remodel the peri-osteocytic matrix and secrete collagen [61], possibly enabling more bone flexibility compared to lamellar bone. It is also interesting that the bone architecture within the internal structure of the scutes may help tolerate environmental stressors. For example, the organization of osteons in different directions provides more strength to the bony tissue that holds pressure and tension from different directions. Overall, the presence of woven bone means the catfish maintains superior flexibility (as woven bone is more flexible than lamellar bone), whilst the presence of lamellar bone contributes towards overall bone strength. These properties increase flexibility to avoid predation and also increase chances of survival following predation [27,54].

Both the connection of the dermal scutes with the inter-scute ligaments and the lepidotrichia and the connection of the hemisegments of the fin spine and rays with the intralepidotrichial ligaments were detected in our study using Mallory’s triple stain. Sharpey’s fibres extended from the ligament and inserted into the bony tissue for proper fixation of the dermal skeleton units with homologies. Inter-scute ligaments may be essential in stabilizing the protective barrier of the dermal skeleton. The intralepidotrichial ligaments link the lepidotrichia and were connected to the fin muscles, which could help synchronize the movement of the body during swimming. Furthermore, the intralepidotrichial ligaments allow sequential movement of the fin rays according to swimming directions. Collagen fibres (Sharpey’s fibres) play a role in scute formation by connecting and anchoring different elements into the dermis. During ossification, the surrounding collagen fibres are incorporated into the bone matrix [19]. The bony structure of the scute, spine, and rays of the dorsal fin were also stained with orange G in the present research. The dye’s strong reactivity to the basic units of type I collagen protein may explain the bone’s high affinity for orange G [62]. Furthermore, because of its small size and low molecular weight, orange G can easily penetrate all tissue structures, then firmly retained in compact tissues like bone matrix [63]. In relation to this, Zhu and collegaues [54] concluded that collagen fibres mitigate the compressive power of the predator’s jaws, and therefore this may be why this structural component, as observed in the present research, is present in the *Plecostomus*.

The present research showed how the microstructure and organization of bone tissue in the dermal skeleton, including the scutes and fins of the common pleco, also differed across anatomical regions. The dermal scutes in *Hypostomus plecostomus* consist of three portions: the central, preterminal, and terminal portions. Each portion was made up of three layers: a superficial bony plate, a basal bony plate, and a mid-plate. The superficial and basal bony plates were composed of lamellar bone and lamellar zonal bone. The mid-plate consists of secondary osteons and woven bone. In the terminal portion, the superficial and basal bony plates became thinner. This benefits the common pleco as protection from predators and environmental surroundings (e.g. particle/rocks in rivers) is given by the lamellar bone on the outer layers, whilst flexibility is provided by the woven bone in the mid-plate. The woven bone also maintains a greater capacity for remodelling and can therefore support the remodelling of the superficial and basal bony plates. The fin consisted of spine and rays that comprise lepidotrichium (two hemisegments), and the spine contained centrifugal and centripetal lamellar and trabecular bones. A centripetal fibrous bone was located between the secondary bones and the hemisegments of the fin rays were V-shaped and composed of thin centrifugal and centripetal lamellar bones and trabecular bones. The fibrous bone was of particular interest as again it appeared as if more collagen we present, potential enabling more flexibility between the bones. Future work could consider integrating the microstructure organization in multiscale finite element modelling of the structures that compose bones, such as that proposed in single osteons, to understand how the damage process at the microscale can affect the mechanical behaviour of bone at the macroscale, as investigated by Gaziano and co-authors [64]. Similarly, it would be interesting to develop more information and modelling about the evolution of fibre distributions in the tissue growth and remodelling, as proposed by Gizzi and co-authors [65], especially as this may open insights into tissue stresses and stiffness to understand tissue movement under situations such as swimming and to develop further concepts into the evolution of continuous fibre distributions under loading, such as when facing predation.

Osteoderms, plate-like bone deposits, vary greatly in size, shape, surface ornamentation, articulation, geometry and functions between and within taxa. The skeletal matrix of osteoderms always includes osseous tissue, as seen in the present *Hypostomus plecostomus* research. Most osteoderms contain varying amounts of mineralized and non-mineralized fibrous connective tissue, and bone marrow [15], as also described in the present research. It is likely that the functions are as diverse as the species with osteoderms as most tetrapod lineages, including amphibians, lepidosaurs, archosaurs, turtles, para-reptiles, placodonts, and even some synapsids, have osteoderms [66–68]. For example, in some crocodiles (American alligators), the highly vascularized osteoderms play a significant role in thermoregulation [69], as a calcium-storage site, are necessary for egg-laying [70], and maintain the acid–base balance, which neutralizes acidosis caused by prolonged immersion in water and thus increases carbon dioxide in circulation [71]. The dermal bone liberates alkaline ions (calcium and magnesium) into the bloodstream, which serve as a buffer and neutralize the acidification of body fluids. Whilst some amphibians such as caecilians lack mineralized dermal scales between the skin’s furrows [72] others have adapted. For example, frogs have dermal bones, and regulate gas exchange through the skin, whereas amniotes (ancestral tetrapods), have thinner dermal scales, and depend on gas exchange through lung ventilation [73]. Tortoises and turtles have dome-shaped, keratin-covered dermal shells [74], serving biophysical functions such as camouflage, waterproofing [75], buoyancy [76], a reservoir for metabolites (such as fat and ions) [77], and mechanical and predator protection [78]. Besides being taxonomically widespread, the phylogenetic distribution of the osteoderms is exceedingly, unpredictable [68,79–82]. The examples above highlight the crucial need for structural data, preferably at the tissue level to pinpoint the exact nature of the similarly shaped dermal skeletal components and try to infer their evolutionary history and functions. The current research provides valuable data that helps understand the microscopic structures, and therefore the evolutionary links between the various parts of the vertebrate dermal skeleton. It can also help identify and classify new species and offer taxonomic detail in cases such as the potentially new *Hypostomus* species found in Brazil [83–86] and support ongoing investigations where there are taxonomic doubts such as in *Hypostomus pantherinus* [87], or provide additional avenues for investigation where there is less information relating to a particular species such as the *Hypostomus variipictus* [88].

## 5. Conclusions

This study aimed to investigate the microstructure and organization of bone tissues of the dermal skeleton of the common pleco (*Hypostomus plecostomus*). This was the first study to investigate the scutes and fins using light microscopy, stereomicroscopy, and scanning electron microscopy alongside differing histological stains. Moreover, we have explored the significance of these structures adapting to the aquatic environment, behaviour, and evolution of the pleco and compared them against other species. We therefore concluded that the shape, direction, organization, microstructure, and histochemical properties of the scute and fins of the common pleco are designed for their environmental adaptation and provide protection against predation. Our research has illustrated the microstructural architecture of the bony scutes, spine, and rays of the bony fins of the common pleco (*Hypostomus plecostomus*). Naturally, this study shows the anatomy and histology of one species, further studies in more species will aid comparative morphology and evolutionary studies, especially in relation to structure and function. We recommend that future studies involving transcriptome analysis be performed, and at different stages in scute formation, to identify the genes contributing towards their formation and for comparative studies to be conducted, to further understand their roles in evolution. We would also recommend that understanding the mechanical stresses, functions and evolution may be supported with advanced mathematical modelling, helping to shed light on the performance of each tissue type and of the structures within the tissues.

## Supplementary Material

Supplemental Material

## Data Availability

The datasets during and/or analysed during the current study available from the corresponding author on reasonable request.
