# Supplementary material for: Microstructural architecture of the bony scutes, spine, and rays of the bony fins in the common pleco (Hypostomus plecostomus)
Source: Int J Vet Sci Med. 2024 Sep 4;12(1):101–24. doi: 10.1080/23144599.2024.2374201 (PMC11376312; doi:10.1080/23144599.2024.2374201)
Supplement: Supplemental Material [file TVSM_A_2374201_SM5686.zip › TVSM_A_2374201_Supplementary.docx]

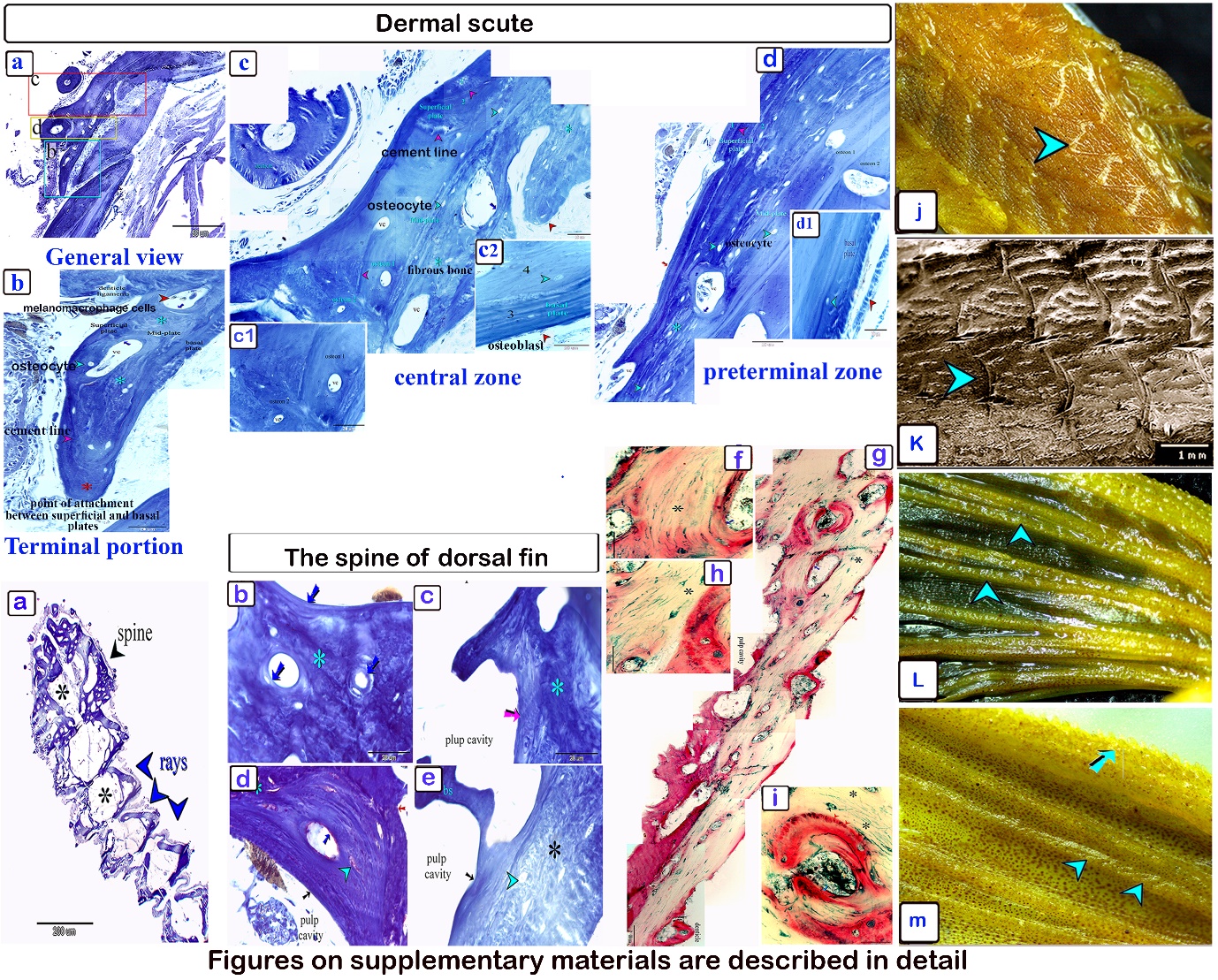


**Graphical Abstract. Gross anatomical and light and scanning electron microscopy investigating into the dermal scute and dorsal fin of the Common Pleco (*Hypostomus Plecostomus*).**

**The histological structure of terminal, central and per terminal portions of the dermal scute**. (a) general view of the dermal scute. (b) Terminal portion, (c) Central portion and (d) per terminal portion.

(b, c, d) The superficial and basal bony plate consisted of two layers (1, 3), the lamellar bone was the most superficial layer which consisted of longitudinally arranged bone lamellae around the vascular channel (vc). The cement line separated the two layers (pink arrowhead). The mid-plate consisted of lamellar bone (and osteons), and the osteons consisted of concentrically arranged bone lamellae encircling vascular chambers (VC). The cement substance (pink arrowheads) distinguished each osteon. (c1) The collagen fibres in the osteons (1 and 2) aligned in a perpendicular direction. Interstitial tissue between the osteons incorporated fibrous or fibro lamellar bone. The fibrous bone (*) had coarse collagen bundles and fibers and fibers arranged in irregular manner. Note the osteocytes (turquoise arrowheads) and osteoblasts (red arrowheads). The lamellar zonal bone was covered by osteoblasts. Where the superficial and the basal bony plates met (red asterisk) they became thinner compared to the other portions in terminal portion.

**The spine of dorsal fin**

(a) Cross-section revealing spine hemi-segments (black arrowheads) and rays (blue arrowheads). (b) Between the secondary bone (blue arrows), woven or fibrous bone was discovered (*). (c) The spine had thin centripetal lamellar bone (pink arrow) and fibrous bone (*). (d) The trabecular bone was formed by bone spicules and fully developed secondary bone was identified between the centrifugal (red arrows) and centripetal (black arrows). Osteocytes (turquoise arrowheads) and the pulp cavity contained nerve fibers. (e) The hemisegments contained centripetal lamellar bone (black arrow) and centrifugal woven or fibrous bone (blue asterisk), Note the vascular channel (vc), and bone spicules (ps) that formed the trabecular bone. (f, h) Lamellar bone (asterisk). Safranin positive bone matrix was identified in the secondary osteons within the mid-plate (blue arrow). (g) Longitudinal sections of the spine. Safranin positive bone matrix was observed in the centrifugal (red arrow), centripetal (black arrow) lamellar bone and in the secondary osteons within the mid-plate (blue arrow). (i) Osteocytes (arrowhead) were present, and the osteoblasts were cuboidal in shape, and were aligned at the margins of the secondary osteons, and secreted Safranin positive bone matrix. Note the lamellar bone (asterisk) and vascular channel (VC).

**Dermal scute and pectrol fin visualized using a stereomicroscope and scanning electron microscope.**

(j and k) The dermal scutes were examined by using stereoscope (k) and SEM (l). The dermal scutes (arrowheads) were pentagonal in shape and were covered by denticles. The scutes aligned obliquely and the caudal portion was directed dorsally. (l and m) Structural features of rays of the pectoral fin using a stereoscope. The pectoral fin was comprised of a single spine that had numerous large declines (arrows) and fin rays covered by a web of skin and bears small denticles (arrowheads).
