# Supplementary material for: Microstructural architecture of the bony scutes, spine, and rays of the bony fins in the common pleco (Hypostomus plecostomus)
Source: Int J Vet Sci Med. 2024 Sep 4;12(1):101–24. doi: 10.1080/23144599.2024.2374201 (PMC11376312; doi:10.1080/23144599.2024.2374201)
Supplement: Supplemental Material [file TVSM_A_2374201_SM5686.zip › supplementary data final new.doc]

**Supplementary data on graphical abstract**

**The histological structure of terminal, central and per terminal portions of the dermal scute**. (a) general view of the dermal scute. (b) Terminal portion, (c) Central portion and (d) per terminal portion.

(b, c, d) The superficial and basal bony plate consisted of two layers (1, 3), the lamellar bone was the most superficial layer which consisted of longitudinally arranged bone lamellae around the vascular channel (vc). The cement line separated the two layers (pink arrowhead). The mid-plate consisted of secondary or mature bone (osteon), and the osteons consisted of concentrically arranged bone lamellae encircling vascular chambers (VC). The cement substance (pink arrowheads) distinguished each osteon. (c1) The collagen fibres in the osteons (1 and 2) aligned in a perpendicular direction. Interstitial tissue between the osteons incorporated fibrous or fibro lamellar bone. The fibrous bone (*) had coarse collagen bundles and fibers and fibers arranged in irregular manner. Note the osteocytes (turquoise arrowheads) and osteoblasts (red arrowheads). The lamellar zonal bone was covered by osteoblasts. Where the superficial and the basal bony plates met (red asterisk) they became thinner compared to the other portions in terminal portion.

**The spine of dorsal fin**

(a) Cross-section revealing spine hemi-segments (black arrowheads) and rays (blue arrowheads). (b) Between the secondary bone (blue arrows), woven or fibrous bone was discovered (*). (c) The spine had thin centripetal lamellar bone (pink arrow) and fibrous bone (*). (d) The trabecular bone was formed by bone spicules and fully developed secondary bone was identified between the centrifugal (red arrows) and centripetal (black arrows). Osteocytes (turquoise arrowheads) and the pulp cavity contained nerve fibers. (e) The hemisegments contained centripetal lamellar bone (black arrow) and centrifugal woven or fibrous bone (blue asterisk), Note the vascular channel (vc), and bone spicules (ps) that formed the trabecular bone. (f, h) Lamellar bone (asterisk). Safranin positive bone matrix was identified in the secondary osteon of the mid-plate (blue arrow). (g) Longitudinal sections of the spine. Safranin positive bone matrix was observed in the centrifugal (red arrow), centripetal (black arrow) lamellar bone and in the secondary osteon of the mid-plate (blue arrow). (i) Osteocytes (arrowhead) were present, and the osteoblasts were cuboidal in shape, and were aligned at the margins of the secondary osteon, and secreted Safranin positive bone matrix. Note the lamellar bone (asterisk) and vascular channel (VC).

**Dermal scute and pectrol fin visualized using a stereomicroscope and scanning electron microscope.**

(j and k) The dermal scutes were examined by using stereoscope (k) and SEM (l). The dermal scutes (arrowheads) were pentagonal in shape and were covered by denticles. The scutes aligned obliquely and the caudal portion was directed dorsally. (l and m) Structural features of rays of the pectoral fin using a stereoscope. The pectoral fin was comprised of a single spine that had numerous large declines (arrows) and fin rays covered by a web of skin and bears small denticles (arrowheads).

**Supplementary Figure 1:** **Negative image the histological structure of the central portion of the dermal scute on the dorsal side of the common pleco head. Semi-thin sections stained with toluidine blue.** (a) General view of the dermal scute. Note the squared area refers to the central portion of the scutes. (b) The superficial bony plate consisted of two layers; the lamellar bone (1) and the lamellar zonal bone (2) between which is demarcated by the cement line (green arrowhead). The lamellar bone was the most superficial layer, which consisted of longitudinal arranged bone lamellae. The lamellar zonal bone had concentrically arranged bone lamellae around the vascular channel (vc) which gave rise several branches. The mid-plate was a thick bony plate which consisted of secondary or mature bone (osteon). The osteon was composed of concentric bony lamellae and surrounded the vascular cavities. Each osteon was delineated by cement substance (green arrowheads). The direction of the collagen fibers in the osteons (1 and 2) were aligned in perpendicular manner. Interstitial tissue between the osteons was occupied by the fibrous or fibro lamellar or pseudo−lamellar bone. This type of bone marked the mid-bony plate of the preterminal portion. The fibrous bone (*) contained coarse collagen bundles and fibers arranged in irregular manner. (c) The basal plates were composed of lamellar bone and a lamellar zonal bone. Note the osteocytes (red arrowheads) and osteoblasts (turquoise arrowheads). The lamellar bone (3) was the most basal layer, which consisted of longitudinal arranged bone lamellae. Lamellar zonal bone (4) was concentrically arranged bone lamellae around the vascular channels. The lamellar zonal bone was covered with osteoblasts. (d) The direction of the collagen fibers in the osteons (1 and 2) aligned perpendicularly.

**Supplementary Figure 2: Negative image of the histological structure of the preterminal portion of the dermal scute on the dorsal side of common pleco head stained with toluidine blue.** (a) General view of the dermal scute. Note the squared area refers to the preterminal portion of the scutes. (b) The Superficial bony plate consisted of two layers; the lamellar bone (1, turquoise arrow) and lamellar zonal bone (2) between which was demarcated by the cement line (green arrowhead). The lamellar bone was the most superficial layer, which consisted of longitudinal arranged bone lamellae. Lamellar zonal bone had concentrically arranged bone lamellae around the vascular channels (vc) which gave rise to several branches. The mid-plate was a thick bony plate which consisted of secondary or mature bone (osteon). The osteon was composed of concentric bony lamellae surrounding the vascular cavities. Each osteon was delineated by cement substance (violet arrowheads). The direction of the collagen fibers in the osteons (1 and 2) aligned in a perpendicular manner. Interstitial tissue between the osteons was occupied by fibrous or fibro-lamellar or pseudo−lamellar bone. This type of bone marked the mid-bony plate of the preterminal portion. The fibrous bone (*) had coarse collagen bundles and fibers arranged in an irregular manner. Note the osteocytes (red arrowheads) and osteoblasts (turquoise arrowheads). (c) The basal plate was composed of lamellar bone, the superficial layer appeared striated (osteoblast canaliculi, white arrow). Note the red arrowhead refers to osteoblast, and osteocytes are shown with turquoise arrowheads.

**Supplementary Figure 3: Negative image of the histological structure of the terminal portion of the dermal scute on the dorsal side of the common pleco head.** Semi-thin section of the dermal scute stained with toluidine blue. (a) General view of the dermal scute. Note the squared area refers to the terminal portion of the scutes. (b) Once the superficial and the basal bony plates met (*), they became thinner compared to the other portions, the mid-plate became thinner. Primary/immature cellular bone containing osteocytes (red arrowhead) were observed between the secondary osteon (yellow arrows). Primary or immature cellular bone exhibited irregular collagen orientation that appeared woven (*). Note the melanomacrophage cell (turquoise arrowheads) and cement line (green arrowhead). (c) The terminal portions of the two adjacent dermal scutes were connected with the fibrous components (*) of the inter-scute ligaments. Note the presence of osteocytes (red arrowhead). (d) The superficial, the basal and the mid- bony plate became thinner compared to the other portions. Note the fibrous bone (*) and the vascular cavity (vc).

**Supplementary Figure 4:** **Negative image of the connections between the dermal denticles and the bony scutes on the lateral side of common pleco head.** Semi-thin sections of the dermal scute (a-c) and fin spines (d-g) stained by toluidine blue. The denticles were formed from dentin (red arrowhead) and enamel (pink arrowhead). The dentine was secreted by odontoblasts (turquoise arrowhead) while the enamel was formed from ameloblast (white arrowhead). The pulp cavity of the denticle was connected to the vascular channels of bony scutes or the spines. The superficial lamellar bone (turquoise asterisk) of the scute formed the pedicle (green asterisk) in which the denticles ligament (red asterisk) was attached to. Inside the pedicle, there were fibroblasts (black arrowhead) that formed the denticles ligament (red asterisk). Note the osteonal lamellae (black asterisks), and woven or fibrous bone (brown asterisk).

**Supplementary Figure 5:** **Negative image of the histological structure of the spine in the dorsal fin of common pleco, semithin sections stained with toluidine blue.** (a) Cross-section of dorsal fin showing hemisegments of the spine (white arrowhead) and rays (yellow arrowheads). Hemisegments of the spine had centrifugal (1) and centripetal (2) trabecular bone. (b) Longitudinal section of the dorsal fin showing the lepidotrichium of the spine (white arrowhead) and rays (yellow arrowheads). Note the pulp cavity (white asterisk). (c) Thin centripetal lamellar bone (green arrow) and the centripetal woven or fibrous bone (*). (d) Thin centrifugal lamellar bone (turquoise arrow), with centrifugal woven or fibrous bone (*), secondary bone (yellow arrows) and a vascular channel (vc). (e, f) The centrifugal woven or fibrous bone (*), secondary bone (yellow arrows) and a vascular channel (vc).

**Supplementary Figure 6: Negative image of** **the histological structure of the rays of the dorsal fin of common pleco - semithin sections stained with toluidine blue.**

(a) Longitudinal section of dorsal fin showing the lepidotrichium (yellow arrows) of the rays (turquoise arrow). Note the v-shaped hemisegments, d = denticle. (b, c) The v-shaped hemisegments had thin centrifugal lamellar bone (turquoise arrow), centrifugal woven or fibrous bone (white asterisk), secondary bone (yellow arrows), and pulp cavity which contained nerve fibers. (d, f) In some areas, the rays of the dorsal fin were formed from centrifugal pseudo- (turquoise arrow) and centripetal (white arrow) lamellar bone. Inactive osteoblasts (turquoise arrowhead) and osteocytes (red arrowhead) were present. (g, h) The v-shaped hemisegments contained thin centrifugal lamellar bone (turquoise arrows), and centrifugal (white arrows), woven or fibrous bone (white asterisk). (e, i) The rays consisted of thin centrifugal (turquoise arrow) and centripetal (white arrow) lamellar bone, between which woven bone (white asterisk) was present. Intralepidotrichial ligaments connected adjacent lepidotrichium. Note, the bone spicule or trabeculae (bs). (j,k) Thin centrifugal (turquoise arrow) and centripetal (white arrow) lamellar bone. In figure (j) the woven or fibrous bone (white asterisk) was oriented in three layers. Intralepidotrichial ligaments connected adjacent lepidotrichium. (l) Intralepidotrichial ligaments continued in the centrifugal bone with Sharpy’s fibers (double red arrows).

Note: In all figures, the osteoblasts (turquoise arrowhead) covered the surface of the centrifugal bone, osteocytes (red arrowhead), secondary bone (yellow arrows), vascular channel (vc), woven or fibrous bone (white asterisk) were identified.

**Supplementary Figure 7:** **Negative image of the histological structure of the dermal scute on the dorsal side of the common pleco head.** H&E-stained paraffin sections. (a) The dermal scute protected the brain. (b, c) The superficial bony plate (turquoise arrow) connected with the denticles (d) via the denticle ligament (turquoise asterisks) which was secreted by fibroblasts (turquoise arrow). The denticle pulp cavity (pc) continued with the vascular channels (vc) of the dermal scutes. The mid plate was formed from trabecular bone that contained fibrous bone (white asterisk) and the secondary osteons (yellow arrow). (d-f) The superficial (turquoise arrow) and basal (white arrow) bony plates and a secondary osteon in the mid plate (yellow arrow).

**Supplementary Figure 8:** **Negative image the histological structure of the spine of the common pleco dorsal fin.** H&E-stained paraffin sections. (a): The dorsal fin consisted of spine and rays. (b, c) The spine had centrifugal and lamellar (green arrow) and trabecular bone (yellow asterix) and centripetal lamellar (turquoise arrow) and trabecular (white asterisk) bone.

**Supplemental Figure 9:** **Negative image of the histological structure of the rays of the dorsal fin in common pleco.** H&E-stained paraffin sections. (a-c, e) The rays of dorsal fin consisted of lepidotrichium which formed of two hemisegments connected by intralepidotrichial ligaments (yellow arrows). Note the denticles (d) attached to the bony tissue of the fin ray. Hemisegments were composed of thin centrifugal (turquoise arrow) and centripetal (white arrow) lamellar bone and trabecular bone (T). (d) The v-shaped hemisegments were composed of thin centrifugal (turquoise arrow) and centripetal (white arrow) lamellar bone and trabecular bone (T) and the two hemisegments were connected by intralepidotrichial ligaments (yellow arrows). Denticles (d) were attached to the bony tissue of the hemisegment.

**Supplementary Figure 10:** **Negative image of the histological structure of the common pleco scute using safranin O-fast green on the lateral side of the fish dorsal fin. (**a) Longitudinal-sections of the scute, with safranin positive bone matrix observed in the superficial (red asterisk), basal (blue asterisk) bony plate and in the secondary osteons of the mid plate (white asterisk). (b, c) Safranin positive bone matrix was observed in the secondary osteons (white asterisk) compared with the primary osteon (white asterisk). Importantly the osteocytes (white arrowhead) had cytoplasmic processes (double white arrowheads) that formed networks within bone matrix. (d) Safranin positive bone matrix was observed in the superficial (turquoise arrow), and basal (white arrow) bony plates underneath the periosteum.

**Supplementary Figure 11:** **Negative image of the histological structure of the spine of the common pleco dorsal fin using safranin O fast green. (**a) Longitudinal-sections of the spine. Safranin positive bone matrix was observed in the centrifugal (turquoise arrow), centripetal (white arrow) lamellar bone and in the secondary osteons in the mid plate (yellow arrow). (b, c) Osteocytes (white arrowhead) had cytoplasmic processes (double white arrowhead) that formed networks within bone matrix. Lamellar bone (white asterisk), vascular channel (vc). (d, e) Lamellar bone (white asterisk). Safranin positive bone matrix was identified in the secondary osteons within the mid plates (yellow arrow). (f:) Osteocytes (white arrowhead) were cuboidal in shape, aligned at the margins of the secondary osteon, and can secrete safranin O positive bone matrix (turquoise arrowheads). Note lamellar bone (white asterisk). (g) Osteon 1 and 2 were organized perpendicular to each other, note the vascular channel (vc).

**Supplementary Figure 12: Negative image of** **the histological structure of the longitudinal view of the hemisegment in the dorsal fin ray of common pleco using safranin O-fast green.** (a, b) Safranin O positive bone matrix hemisegments were located in the centrifugal (turquoise arrow) and centripetal (white arrow) lamellar bone and trabecular bone (T). Note the denticles (d). © Safranin O positive bone matrix was also identified in the centrifugal (red arrow) and centripetal (white arrow) lamellar bone while weak affinity for safranin O was detected in the deeper lamellar bone (red asterisk).

**Supplementary Figure 13: Negative image of** **the histological structures of the dorsal fin spine and scute of common pleco fish using PAS.** Spine of the dorsal fin (a-g) and dermal scute (h, i). (a-c) Longitudinal-sections with PAS positive matrix of the centrifugal (turquoise arrow), centripetal (white arrow) lamellar bone. Note intralepidotrichial ligaments. (d) Sharpy’s fibers (red arrow) continued alongside the intralepidotrichial ligaments. (e, f) PAS positive bone matrix (yellow arrows) was observed around the vascular channels (vc) and the secondary osteons (pink arrow). (g) Osteocytes (white arrows) were located in lacunae in the lamellar bone. (h, i) All layers of the scute were PAS positive. Note the denticles (d) and inter-scute ligaments (white asterisks).

**Supplemental Figure 14:** **Negative image of the histological structure of the scute on the lateral side of the common pleco head using the Mallory’s triple stain.** Paraffin sections of the dermal scute (a-c) and fin rays (d, e). (a-c) Longitudinal-sections of the scute with inter-scute ligaments connected the dermal scutes. The scutes had a high affinity for orange G and acid fuschin. Note the superficial (turquoise arrows) and basal (white arrows) bony plates and in the secondary osteon in the mid plate (yellow arrow). Denticles (d) were connected to the scute by denticle ligaments (dl). Collagen fibers, which emerged from the inter-scute ligaments, penetrated the scute and formed Sharpy’s fibers (turquoise arrowhead). (d, e) Longitudinal paraffin sections of hemisegment of the fin ray. The centrifugal (turquoise arrow) and centripetal (white arrow) lamellar bone and trabecular bone (T) of the fin rays had high affinity for orange G and acid fuschin. Note, the turquoise arrowhead referring to osteoblast and red arrowhead referring to osteocytes.

**Supplementary Figure 15:** **Negative image of the histological structure of the pectoral fin of common pleco fish demonstrated with Crossman's trichrome.**

Paraffin sections of the scute (a-c) and the spine of the pectoral fin (d-g). (a-c) Longitudinal-sections of the scute. The superficial plate (turquoise arrows) had high affinities for orange G while the mid (white asterisk) and basal plates (white arrows) had high affinities for light green. (d) Longitudinal-sections of the spine. The centrifugal (red arrow) lamellar bone had strong affinities for orange G and acid fuchsin, while the centripetal (black arrow) lamellar bone and trabecular bone (asterisk) had a high affinity for light green. (e,g) The intralepidotrichial ligaments continued as sharpy’s fibers (White arrows). The centrifugal (turquoise arrow) lamellar bone had strong affinities for orange G and acid fuchsin while the trabecular bone (white asterisk) had a high affinity for light green.

**Supplementary Figure 16: Negative image of** **the histological structure of the spine of the dorsal fin in common pleco fish – silver stain.** (a-f): Longitudinal sections of the bony scute identifying the different layers of the bony scutes: the superficial plate (turquoise arrows) and basal plates (white arrows). The mid-plate consisted of lamellar bone (turquoise asterisk), woven bone (white asterisk), and secondary osteon (yellow arrow). Note the denticles (d) exhibited striations of the dentinal tubules.

**Supplementary Figure 17: Negative image showing identification of the surface features of the bony scutes in common pleco fish.** Stereoscope (a-f) and SEM (g, h) showing the dermal scutes (red arrowheads) which were organized in a pentagonal shape and were covered by denticles (red arrowheads). The scutes aligned obliquely that the caudal potion directed dorsally. (a, b, c) Represent the dorsal side of head, (d) represents the lateral side of fish, while (e and f) represent the caudal peduncle. Stereoscope image magnifications: (a-e) 6.3x; (f) 12.5x.

**Supplemental Figure 18: Negative image identifying the fibrillary organization of the lamellar, lamellar zonal bone, and osteonal lamellae of the scute using SEM on the lateral side of the common pleco fish.** (a-e) Lamellar (golden colored), lamellar zonal bone (green colored) comprised of the superficial and the basal boney plates of the scutes. The mid-plate (green colored) contained secondary osteons (red asterisks). Note denticles (gray colored spikes). (f) The basal boney plates of the scutes comprised of the lamellar and the lamellar zonal bone were separated by the demarcating the cement line (green colored).

**Supplementary Figure 19:** **Negative image of scanning electron microscopy identification of vascular channels and fibrous bone in the common pleco scute.** (a-c) Lamellar, lamellar zonal bone (golden colored) comprised the superficial and the basal boney plates of the scutes. The mid-plate (green colored) contained secondary osteons (red asterisks). Note the osteogenic cells (pale blue colored) located in the vascular channels and the fibrous bone (blue asterisk). (d) Osteogenic cells (light blue colored) were located in the vascular channel of the basal plate. Note the osteocytes (blue arrowhead) had a flattened profile and multiple cell processes.

**Supplementary Figure 20:** **Negative image of the stereoscope structure features of the pectoral fin rays in the common pleco.** The pectoral fin was composed of single spine that had numerous large declines (red arrows) and fin rays covered by web of skin and bears small denticles (red arrowheads). Magnification of stereo images: (a) 6.3x; (b) 12.5x; (c) 40x.

**Supplementary Figure 21: Negative image of the scanning electron microscopy structure of pectoral fin rays in the common pleco fish.** (a, b) Two Hemisegments (golden colored) formed lepidotrichium which attached to the intralepidotrichial ligaments (green colored). Note the trabecular bone (red asterisks) and denticles (light blue color).

| **Components** | **Crossmon’s trichrome stain** | |
| --- | --- | --- |
| **Solution A: Acid fuchsin Orange G** | Acid fuchsin | 1 g |
|  | Orange G | 400 mg |
|  | Distilled water | 300 ml |
|  | Glacial acetic acid | 3 ml |
| **Solution B: (freshly prepared)** | Phosphomolybdic or phosophtungsticacid | 1 g |
|  | Distilled water | 100 g |
| **Solution C: Light green** | Light green | 1 g |
|  | Distilled water | 100 ml |
|  | Glacial acetic acid | 1 ml |
| **Solution D: Glacial acetic acid** | Glacial acetic acid | 1 ml |
|  | Distilled water | 99 ml |
| **Method** | 1-Deparafinization in xylene I and II for 20 minutes and hydrate in ethanol alcohol 100, 90, 80, 70% for 5 min in each concentration.  2- Rinse in distilled water  3- Rinse in distilled water stain in acid fuchsin Orange G for 1-5minutes  4- Rinse in a distilled water  5- Differentiate in 1% phosphomolybdic acid 5-10 minutes (until the red colour disappear and the connective tissue becomes white).  6- Rinse in distilled water. Differentiate in 1% watery acetic acid (1 ml acetic acid+ 99 D.W) (until the green colour disappears from the muscles).  7- Stain in light green 1% for 5 minutes and then rinse in distilled water.  8- Differentiate in 1%watery acetic acid (until the green colour disappears from the muscles).  9- Rinse in distilled water.  10- Dehydrate 3 times in 96% alcohol.  11- Dehydrate in 100% alcohol and then clear with xylene I, II  (Muscles become red, connective tissue becomes green and the nucleus becomes red).  12- Clear with xylene and cover using DPX. | |

Supplementary Table 1: Components and procedures for Crossomon’s trichrome

| **Components** | Mallory trichrome stain | |
| --- | --- | --- |
| **Solution A: Acid fuchsin** | Acid fuchsin | 1 g |
|  | Distilled water | 100 ml |
| **Solution B: (freshly prepared)** | Phosphomolybdic or phosophtungsticacid | 1 g |
|  | Distilled water | 100 g |
| **Solution C: Light green** | Orange G | 1 g |
|  | Methyl blue | 0.5 g |
|  | Oxalic acid | 2 g |
|  | Distilled water | 100 ml |
| **Method** | 1-Deparafinization in xylene I and II for 20 minutes and hydrate in ethanol alcohol 100, 90, 80, 70% for 5 min in each concentration.  2- Rinse in distilled water  3. Soak for 2 minutes in solution A.  4. Rinse well with distilled water.  5. Soak for 2 minutes in solution B.  6- Rinse in distilled water and left for dry  7- Dehydrate in 96% alcohol for 1 min.  8- Dehydrate in 100% alcohol for 1 min and left for dry and then clear with xylene I, II  9- Cover using DPX. | |
|  | Expected results   - Nuclei  –  red - Erythrocytes  –  orange - Muscle  –  red - Collagen  –  blue | |

**Supplementary Table 2: Components and procedures of Mallory's Trichrome stain**

| **Components** | Safranin O Staining | |
| --- | --- | --- |
| **Solution A:**  **0.05% Fast Green (FCF) Solution** | Fast Green | 0.05 g |
|  | Distilled water | 100 ml |
| **Solution B**:  **1% Acetic Acid Solution** | Glacial Acetic acid | 1 ml |
|  | Distilled water | 99 ml |
| **Solution C:**  **0.1% Safranin O Solution:** | Safranin O | 0.1 g |
|  | Distilled water | 100 ml |
| **Method** | 1-Deparafinization in xylene I and II for 20 minutes and hydrate in ethanol alcohol 100, 90, 80, 70% for 5 min in each concentration. Rinse in distilled water.  2- Wash in running tap water for 10 minutes.  3-   Stain with fast green solution for 5 minutes.  4-   Rinse quickly with 1% acetic acid solution for no more than 10 –15 seconds.  5-   Stain in 0.1% safranin O solution for 5 minutes and left to dry.  6-   Dehydrate with 95% ethyl alcohol, absolute ethyl alcohol, left for dry then clear with xylene, using 2 changes each, 2 minutes each.  7- Cover using DPX. | |

**Supplementary Table 3: Components and procedures of safranin O staining with fast green stain**

| **Components** | Grimelius silver nitrate | |
| --- | --- | --- |
| **Solution A:**  **S Silver solution:**  **1% aqueous silver nitrate** | Silver nitrate | 1 g |
|  | Distilled water | 100 ml |
| **Solution B:** | Acetate buffer (pH 5.6) | 10 ml |
|  | Distilled water | 87 ml |
| **Preparation of acetate buffer** | Stock A→ 1.2 ml glacial acetic acid+ 100 ml D.W.  Stock B→ 1.64 g sodium acetate anhydrous+ 2.72 g sod.  Acetate tri hydrate + 100 ml D.W.  Mix 9.6 ml from stock A with 90.4 ml from stock B. | |
| **Solution C:**  **Reducing solution** | Hydroquinone | 1 g |
|  | Sod. Sulphite crystals | 5 g |
|  | D.W. | 100 ml |
| **Method** | 1-Deparafinization in xylene I and II for 20 minutes and hydrate in ethanol alcohol 100, 90, 80, 70% for 5 min in each concentration. Rinse in distilled water.  2-Place the sections in preheated silver at 60◦c for overnight.  3-Remove sections and drain thoroughly then place it at 65°C for 6 min in freshly prepared reducing solution.  4-Rinse sections in D.W for 1 min.  5-Wash thoroughly in tap water.  6- Dehydrate with 95% ethyl alcohol, absolute ethyl alcohol, and then clear with xylene, using 2 changes each, 2 minutes each.  After the reduction stage, the sections are impregnated twice; treated with 5% sodium thiosulfate (5 g sodium thiosulfate /100 ml DW(, rinsed in D.W at 60°C for 1 minute, then placed in silver solution at 60°C for 15 minutes, followed by a reducing solution at 45°C for 4 minutes. | |

**Supplementary Table 4: Grimelius silver nitrate method**

Supplementary table of sample: Ten common pleco fish (*H. plecostomus*) were separated into the following groups.

| Number of fish | Fixation | Proscessing | cutting | Staining |
| --- | --- | --- | --- | --- |
| Three whole fish | 1-Wrobel–Moustafa fixative for 24 h  2- then the samples were decalcified for 6 weeks, with solution changes every 5 days | The decalcified and fixed specimens were dissected into three parts, then processes then dehydrated in an ascending sequence of ethanol After clearing in three xylene baths, the samples were embedded in melted paraplast | The paraffin blocks were sectioned longitudinally and transversely into 4–5µm thick sections | The following conventional histological and histochemical stains were used to identify structures: Harris’ hematoxylin and eosin (H&E) stain; periodic acid–Schiff (PAS); Crossomon’s trichrome stain Mallory’s triple trichrome stain a mixture of safranin O/fast green; Van Gieson stain and a Grimelius silver nitrate stain |
| three fish were used  for scanning electron microscopy |  |  |  |  |
| and two fish were used for semi-thin section examinations | Small pieces of tissue (2.0–3.0 mm in length) were collected from different areas of the skin and fixed in Karnovsky fixative | Thereafter, decalcification of sections was performed at 4°C using 10% EDTA solution was changed every five days. After decalcification, the sections were washed four times for 15 min in 0.1 M sodium phosphate buffer saline (pH 7.2; The sections were then processed according to the methods previously described [40,47] | (1 µm) sections cut with an ultra-microtome Ultra Cut E | Toluidine blue[41] [sodium tetraborate (borax) 1 g, toluidine blue 1 g, and distilled water 100 mL] was used to stain semi-thin |
| Two fish were used for stereo-microscopical examinations | fixed in Bouin’s fixative solution | immersed in 70% ethanol alcohol and photographed under a binocular stereomicroscope |  |  |
